# Exploration of blood−derived coding and non-coding RNA diagnostic immunological panels for COVID-19 through a co-expressed-based machine learning procedure

**DOI:** 10.3389/fimmu.2022.1001070

**Published:** 2022-11-03

**Authors:** Mohadeseh Zarei Ghobadi, Rahman Emamzadeh, Majid Teymoori-Rad, Elaheh Afsaneh

**Affiliations:** ^1^ Department of Cell and Molecular Biology and Microbiology, Faculty of Biological Science and Technology, University of Isfahan, Isfahan, Iran; ^2^ Department of Virology, School of Public Health, Tehran University of Medical Sciences, Tehran, Iran; ^3^ Department of Physics, University of Isfahan, Hezar Jarib, Isfahan, Iran

**Keywords:** SARS- CoV-2, COVID-19, innate immune pathways, machine learning, WGCNA, vitamin D

## Abstract

Severe acute respiratory syndrome coronavirus 2 (SARS- CoV-2) is the causative virus of the pandemic coronavirus disease 2019 (COVID-19). Evaluating the immunological factors and other implicated processes underlying the progression of COVID-19 is essential for the recognition and then the design of efficacious therapies. Therefore, we analyzed RNAseq data obtained from PBMCs of the COVID-19 patients to explore coding and non-coding RNA diagnostic immunological panels. For this purpose, we integrated multiple RNAseq data and analyzed them overall as well as by considering the state of disease including severe and non-severe conditions. Afterward, we utilized a co-expressed-based machine learning procedure comprising weighted-gene co-expression analysis and differential expression gene as filter phase and recursive feature elimination-support vector machine as wrapper phase. This procedure led to the identification of two modules containing 5 and 84 genes which are mostly involved in cell dysregulation and innate immune suppression, respectively. Moreover, the role of vitamin D in regulating some classifiers was highlighted. Further analysis disclosed the role of discriminant miRNAs including miR-197-3p, miR-150-5p, miR-340-5p, miR-122-5p, miR-1307-3p, miR-34a-5p, miR-98-5p and their target genes comprising *GAN*, *VWC2*, *TNFRSF6B*, and *CHST3* in the metabolic pathways. These classifiers differentiate the final fate of infection toward severe or non-severe COVID-19. The identified classifier genes and miRNAs may help in the proper design of therapeutic procedures considering their involvement in the immune and metabolic pathways.

## Introduction

Coronavirus disease 2019 (COVID-19), a respiratory illness caused by severe acute respiratory syndrome coronavirus 2 (SARS-CoV-2), has lately become an epidemic (1). SARS-CoV-2 infection activates innate and adaptive immune responses. The infected patients show different symptoms from mild to severe, in which host inflammatory responses and virus-specific factors have critical roles (2, 3). Lymphocytopenia is a prevalent condition along with the reduced percentage of eosinophils, monocytes, and basophils as well as a decrease in the numbers of B cells, cytotoxic T lymphocytes known as CD8+ T cells, T helper cells known as CD4+ T cells, and natural killer (NK) cells (1, 4, 5).

The association between the severity of disease with the unusual amount of some pro- or antiinflammatory chemokines, cytokines, and other mediators has been reported (4. 6–8). The higher levels of granulocyte colony-stimulating factor (G-CSF), Interleukin-2 (IL-2), Interleukin-7 (IL-7), Interleukin-10 (IL-10), Monocyte Chemoattractant Protein-1 (MCP-1)/C-C Motif Chemokine Ligand 2 (CCL2), C-X-C motif chemokine ligand 10 (CXCL10)/interferon-gamma-induced protein-10 (IP-10), Tumor necrosis factor-α (TNF-α), and Macrophage Inflammatory Proteins -1 α (MIP-1α)/C-C Motif Chemokine Ligand 3 (CCL3) were detected in the patients who needed ICU admission. Moreover, an increased Interleukin-6 (IL-6) level was found in the dead patients (7, 9–11). The comprehensive investigation of the COVID-19 pathogenesis may lead to the recognition of prognostic biomarkers and the design of targeted therapies.

Differential expression and co-expression analyses are two main approaches that are widely used to identify the significant genes involved in a specific condition such as cancers or infectious diseases (12–16).

However, the genes obtained from analyses are unable to classify the classes present in the data set (17). Therefore, developing a powerful algorithm to find biologically significant genes with an eminent classification precision is advantageous.

Support vector machine (SVM) is a popular supervised classification method. SVM applies kernel functions to carry out classification on non-linear data. It can also be employed to select features in association with the recursive feature elimination (RFE) approach (18). The prior treatment of data may help find more accurate gene classifiers that also have critical functions in the disease progress (19).

In this study, we proposed a pipeline to find the significant gene classifiers that may also have critical roles in the progression of COVID-19. To this end, the differential expression genes (DEGs) and weighted-gene co-expression (WGCN) analyses were used to find the genes relevant to COVID-19 development. Afterward, the identified gene groups were used as input for the RFE-SVM algorithm, and robust and accurate classifier genes were found. Finally, the function of these genes was biologically discussed.

## Materials and methods

### Dataset collection and preprocessing

We downloaded six RNAseq datasets from the Gene Expression Omnibus (GEO) depository comprising 392 samples derived from whole blood or peripheral blood mononuclear cells (PBMCs). We divided the datasets into train and test groups, so that GSE157103 (20), GSE155454 (21), GSE152641 (22), GSE161731 (23) were put in the train group as well as GSE166424 (24) and GSE152418 (25) in the test group. The details of the datasets are described in **Table 1**. A total of 288 and 75 patient and healthy samples, respectively, were placed in the train set; and 52 and 19 patient and healthy samples, respectively, in the test set. We mapped the Ensemble identifiers in each dataset to Entrez gene identifiers to simplify the integration analysis. We also considered technical heterogeneity as the datasets were profiled by various manufacturers. We applied “ComBat_seq” in the sva package executed in the R environment to remove batch effects among different datasets (26). We filtered out the genes with an average count of less than three and the ones with a standard deviation lower than the first quartile of the standard deviation of all genes. We resolved the within-group outlier by removing the samples that had a less average Pearson correlation with other samples than the first quartile of Pearson correlations between all sample pairs. As a result, 321 training samples (64 healthy and 257 COVID-19) were selected for further analysis. We finally normalized the count data using the TMM method (27) executed in the edgeR package

**Table 1:** Details of the datasets included in the train and test analysis.

	Dataset	Platform	Number of samples
**Train**	GSE157103	Illumina NovaSeq 6000 (Homo sapiens)	Healthy: 26 Covid-19: 100
	GSE155454	Illumina HiSeq 4000 (Homo sapiens)	Healthy: 6 Covid-19: 52
	GSE152641	Illumina NovaSeq 6000 (Homo sapiens)	Healthy: 24 Covid-19: 62
	GSE161731	Illumina NovaSeq 6000 (Homo sapiens)	Healthy: 19 Covid-19: 74
**Test**	GSE166424	Illumina HiSeq 4000 (Homo sapiens)	Healthy: 2 Covid-19: 36
	GSE152418	Illumina NovaSeq 6000 (Homo sapiens)	Healthy: 17 Covid-19: 16

A total of 288 and 75 patient and healthy samples, respectively, were placed in the train set; and 52 and 19 patients and healthy samples, respectively, in the test set.

(28) and used the log-transformed normalized count table for further analysis.

A total of 11560 common genes were involved in the analyses. In order to survey the severe and non- severe COVID-19 conditions, the RNAseq datasets including GSE152418 (25), GSE171110 (29), and GSE178967 as well as miRNA dataset GSE176498 (30) were also downloaded from GEO. These datasets contain the RNAseq data related to healthy as well as severe and non-severe (mild to moderate) COVID-19 samples. The details of these datasets are explained in **Table 2**. After performing batch effect and filtering out samples, a total of 102 non- severe and 40 severe samples were considered for the train set. And 49 non- severe and 20 severe samples were put in the test set. The miRNA dataset included 18 non-severe and16 severe specimens.

**Table 2:** Details of the datasets related to the severe and non-severe Covid-19.

Dataset	Platform	Number of samples
GSE171110 (mRNA)	Illumina HiSeq 2500 (Homo sapiens)	severe Covid-19: 44
GSE178967 (mRNA)	Illumina NovaSeq 6000 (Homo sapiens)	severe Covid-19: 12 non-severe Covid-19: 160
GSE152418 (mRNA)	Illumina NovaSeq 6000 (Homo sapiens)	severe Covid-19: 8 non-severe Covid-19: 4
GSE176498 (miRNA)	NextSeq 550 (Homo sapiens)	severe Covid-19: 16 non-severe Covid-19: 18

After removing batch effect and filtering out samples, 102 non- severe and 40 severe samples were considered for the train set; 49 non- severe and 20 severe samples were put in the test set. The miRNA dataset included 18 non-severe and16 severe specimens.

### Hybrid feature selection framework

**Figure 1:**
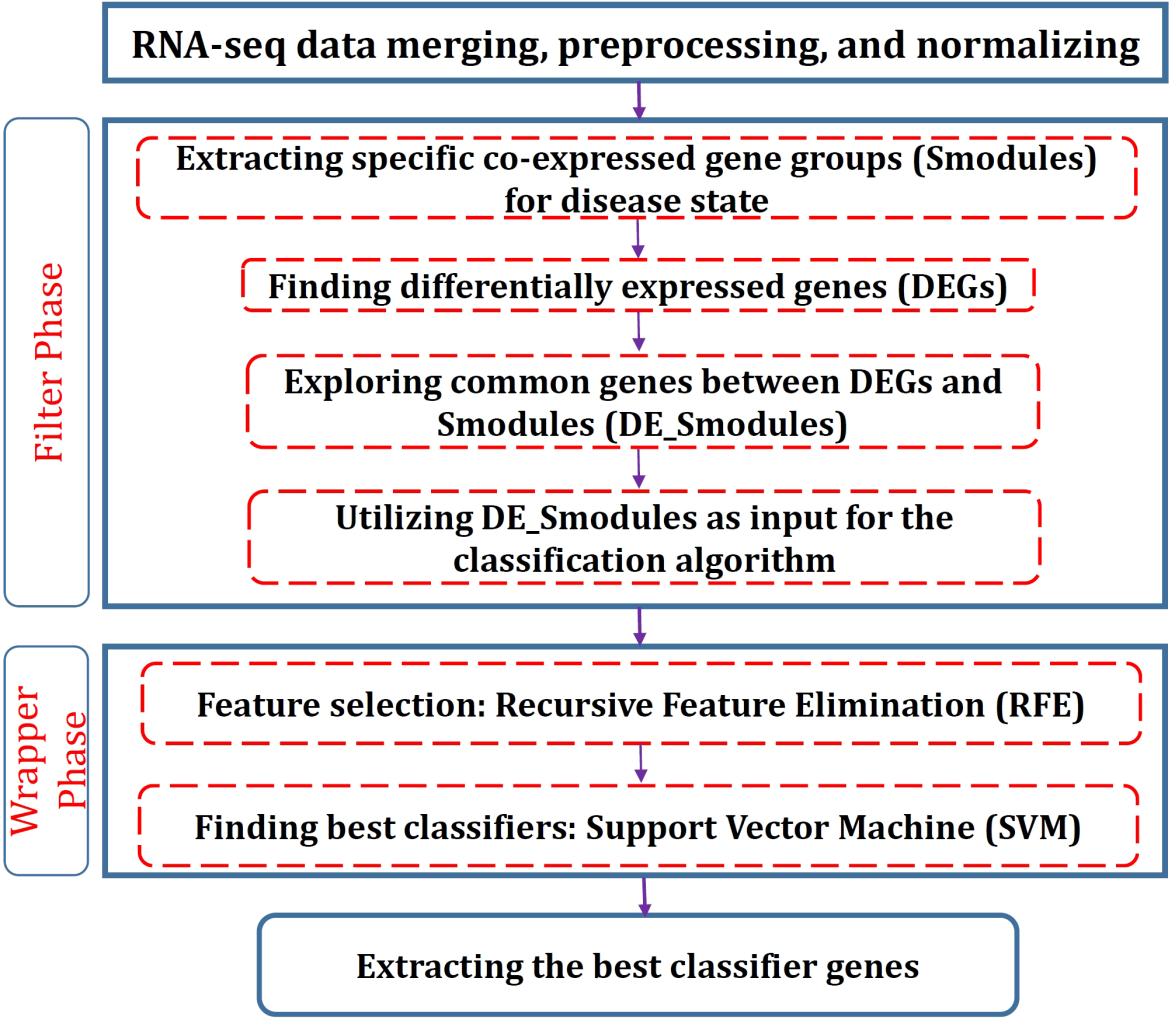
Flowchart of the proposed procedure in this study.

To determine the significant genes, a hybrid method consisting of filter and wrapper approaches is proposed. The filter segment includes two stages: Identification of the co-expressed gene groups (modules) and DEGs. The significantly correlated modules with disease conditions are firstly identified and then the genes that are also among DEGs are found for each module. Eventually, RFE-SVM is employed for the wrapper phase to identify the foremost gene groups with special biological functions as the classifiers. The flowchart of the proposed procedure is depicted in **Figure 1**. In the following, the methods used for each stage are described.

### Weighted gene co-expression network analysis

In order to determine the co-expressed genes (modules), we used weighted gene co- expression network analysis utilizing the WGCNA package in R (31). To this end, a similarity matrix comprising Pearson correlation among all gene pairs was initially created. Next, the soft-thresholding power β was determined by considering scale-free topology fit index 0.8. The weighted adjacency matrix was thereupon calculated by enhancing the elements of the similarity matrix to the soft-thresholding power β and adopting the parameters as follows: type = “signed”, corFnc = “bicor”. Afterward, a topological overlap matrix (TOM) containing the connectivity amount of the gene network was constructed. The dynamic hybrid tree cutting algorithm and “hclust” function were employed to produce the modules by cutting the hierarchical clusters. Finally, the close clusters were merged and ultimate gene groups were identified. Moreover, the modules that were preserved in the external dataset (test set) were determined through module preservation analysis. For this purpose, the “modulePreservation” function in the WGCNA package, as well as permutation-based statistics to find Zsummary and medianRank scores, were employed. Herein, a module with Zsummary <2 and medianRank >8 was defined as non-preserved, a module with 2< Zsummary ≤ 8 and medianRank <8 was interpreted as moderate-preserved, and a module with Zsummary >10 and medianRank <8 was considered as highly-preserved (32, 33).

### Determination of differentially expressed genes

To determine the differential expression of genes, the Bioconductor package DESeq2 was employed (34).

The statistically significant DEGs were detected by applying Benjamini-Hochberg adjusted p-value (35) cutoff of less than 0.05.

### Recursive feature elimination- support vector machine

To pick out the optimal features with a higher discriminative power, RFE-SVM was executed based on tenfold cross-validation (RFECV- SVM). RFE-SVM is fundamentally a backward elimination method. However, the top-ranked variables are not essentially the features that are most relevant. They are actually the most related conditional on the particular ranked subset in the model (18). Top-ranked variables that are excluded in the last iteration of RFE-SVM are the most significant, while the bottom-ranked variables are the least informative and are excluded in the initial iteration (35). RFECV- SVM includes five stages: (i) training SVM on the training dataset based on tenfold cross-validation; (ii) computing ranking criteria based on the calculated SVM weights; (iii) removing features with the smallest ranking criteria; (iv) updating dataset based on the selected features and repeating the process; (v)

feeding the subset of the optimal selected features into the SVM classifier to evaluate the discriminative performance. Eventually, the optimal variable subset with superior discriminative performance is chosen (36, 37). The codes were written in Python 3.

### Pathway enrichment analysis

In order to explore the biological pathways enriched by significant classifier genes, the ToppGene webtool (38) was employed. For this purpose, the ToppFun tool which identifies functional enrichment of input genes according to transcriptome was utilized.

The top and most related pathway terms with a q-value FDR Benjamini-Hochberg <0.05 were considered for further interpretations.

## Results

### Identification of co-expressed gene modules and DEGs

**Figure 2:**
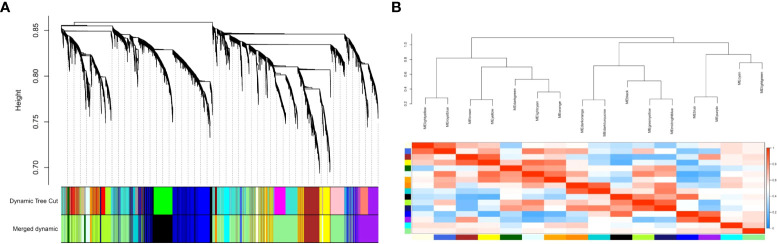
**(A)** The cluster dendrogram and modules before and after merging. The branches of the dendrogram cluster demonstrate the compact interconnected and also highly co-expressed genes; **(B)** The merged modules and their correlations. Each color demonstrates an individual module.

**Figure 3:**
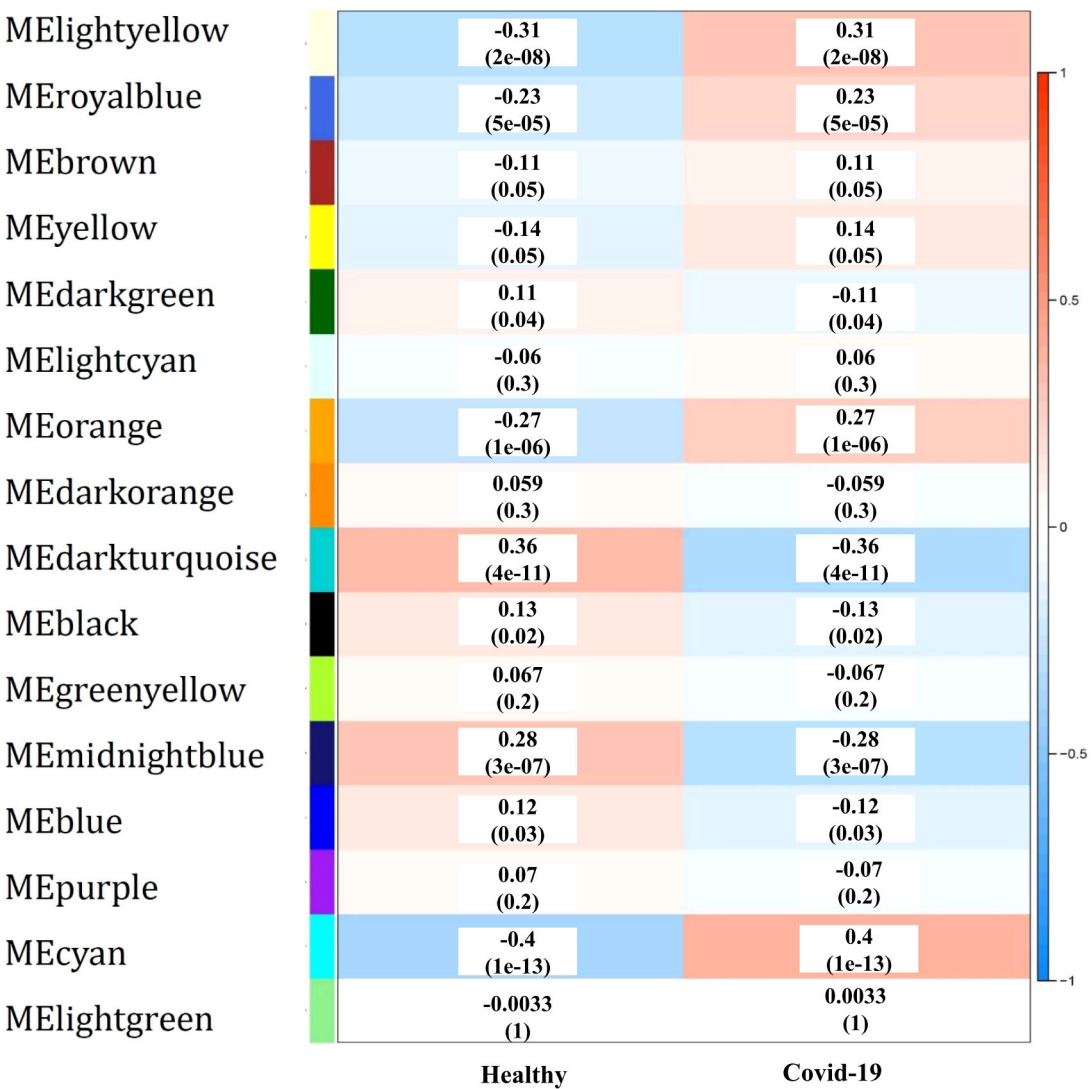
The module-trait relationships, in which the correlation and p-value between modules (vertical axis) and each condition (horizontal axis) are specified.

To identify the co-expressed gene groups related to COVID-19, the weighted gene co- expression network was built. The power β= 3 was acquired as the optimal soft-thresholding power. After calculating adjacency and TOM matrixes and thereupon genes clustering, the neighbor clusters (modules) were merged by adjusting the threshold value to 0.25. As a consequence, 16 modules were resulted (**Supplementary Table 1**). Moreover, **Figure 2A** illustrates the cluster dendrogram and modules before and after merging. The branches of the dendrogram cluster demonstrate the compact interconnected and also highly co- expressed genes. **Figure 2B** indicates the identified modules and their correlations. The unparalleled colors show the individual modules. Next, the module-trait analysis was utilized to identify the significant correlation between modules and COVID-19 (39). **Figure 3** shows the module-trait relationships in which the vertical axis indicates the module names and the horizontal axis indicates different conditions. The p-value<0.05 and correlation>0.25 specifies the modules that considerably are correlated with healthy or COVID-19 conditions. From this analysis, modules including cyan, darkturquoise, lightyellow, midnightblue, and orange were detected as modules with a remarkable correlation with COVID-19 (Smodules). The module preservation analysis revealed that all the mentioned modules were preserved in the external test dataset (**Supplementary Table 2**).

**Figure 4:**
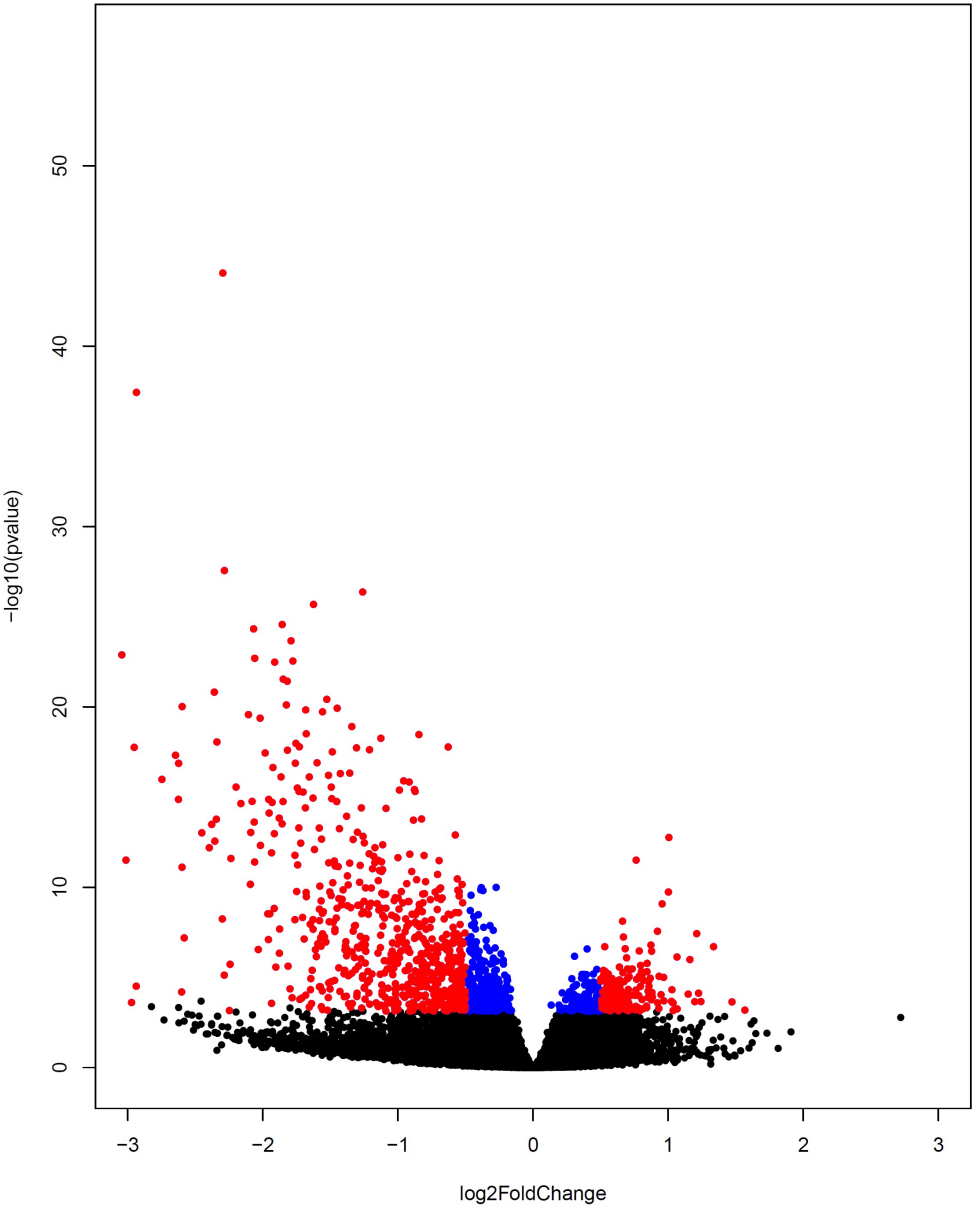
Volcano plot representing differential gene expression between COVID-19 and healthy subjects. The up-regulated and down-regulated DEGs are depicted by colors of red and blue, respectively. The non-DEGs are specified by the black color.

In the next stage, 2203 genes were identified as differentially expressed genes considering Benjamini-Hochberg adjusted p-value < 0.05 (**Supplementary Table 3**), of which 1481 genes were down-regulated (blue color) and 722 were up-regulated (red color) as visualized in the volcano plot (**Figure 4**). To determine the co-expressed genes that were also determined as DEGs, the common genes between DEGs and genes in Smodules were identified (DE_Smodules) (**Supplementary Table 4**).

### Performance analysis using RFECV-SVM analysis

**Figure 5:**
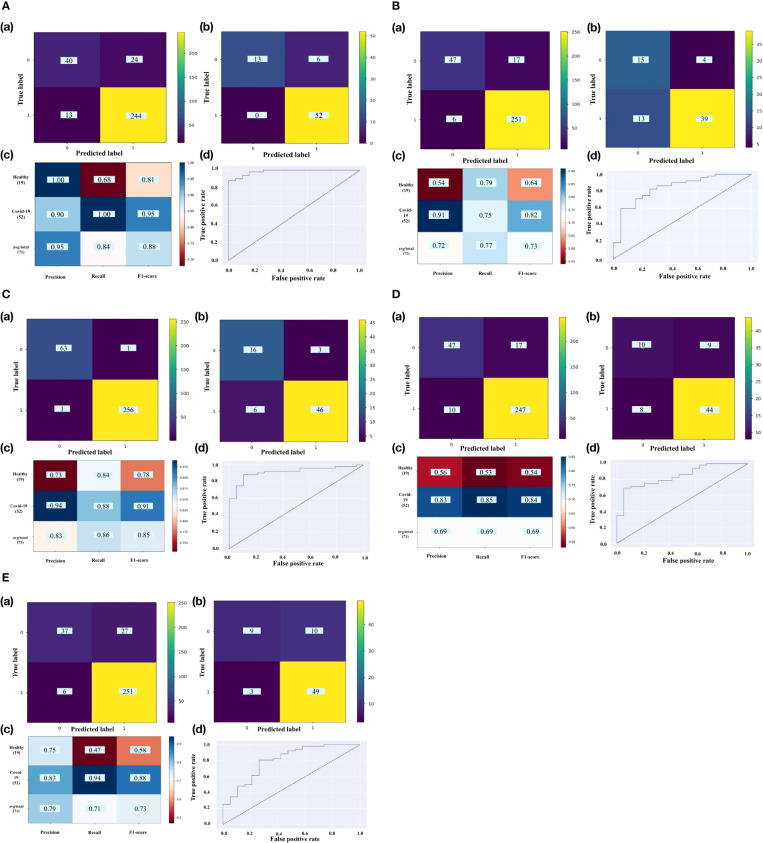
The confusion matrix of **(a)** train set and **(b)** test set; **(c)** classification report; **(d)** ROC curve for DE_Smodules of **(A)** cyan, **(B)** darkturquoise, **(C)** lightyellow, **(D)** midnightblue, **(E)** orange. The confusion matrix shows the number of true and predicted label in healthy (label 0) and COVID-19 (label 1) subjects. The classification report demonstrates the precision, recall, and F1-score for each group.

The RFECV-SVM analysis was applied to compute the obtained accuracy when DEGs, Smodules, and DE_Smodules were used as train data. To validate our proposed hybrid approach, external datasets (test sets) were employed. For this purpose, our proposed method was used to evaluate the accuracy degrees. The accuracy results are mentioned in **Table 3**. **Figures S1-S5** demonstrate the confusion matrixes, classification reports, and ROC curves for Smodules. **Figures 5A–E** also show similar plots for DE_Smodules (common genes between the modules and DEGs). The confusion matrix shows the number of true and predicted labels in healthy (label 0) and COVID-19 (label 1) subjects. The classification report demonstrates the precision, recall, and F1-score for each group. We considered modules that have an accuracy higher than 0.85 for both train and test sets. The highest accuracy degrees and classification parameters including precision, recall, and F1-score were acquired for modules of cyan and lightyellow. The obtained classier genes for these two modules are mentioned in **Supplementary Table 5**. Therefore, these genes could be considered promising biomarkers and therapeutic targets for COVID-19.

**Table 3:** Accuracy of train and test sets; and number of features when genes in Smodules and DE_Smodules were used.

Data	Accuracy (Smodules)	Accuracy (DE_Smodules)
cyan	Train: 0.863 (0.063) Test: 0.89 Number of features: 2	Train: 0.859 (0.065) Test: 0.92 Number of features: 5
darkturquoise	Train: 0.800 (0.123) Test: 0.72 Number of features: 15	Train: 0.832 (0.065) Test: 0.76 Number of features: 45
lightyellow	Train: 0.881 (0.057) Test: 0.80 Number of features: 30	Train: 0.881 (0.046) Test: 0.87 Number of features: 84
midnightblue	Train: 0.782 (0.059) Test: 0.73 Number of features: 33	Train: 0.785 (0.033) Test: 0.76 Number of features: 43
orange	Train: 0.835 (0.054) Test: 0.80 Number of features: 16	Train: 0.804 (0.034) Test: 0.82 Number of features: 20

### Gene enrichment analysis

The pathway enrichment analysis for the identified classifier genes in two groups was carried out. The results disclosed that the genes belonging to the cyan module are involved in “Unwinding of DNA”, “NAD+ metabolism”, “CDK Regulation of DNA Replication”, “Protein export”, “DNA replication”, “Activation of ATR in response to replication stress”, and “Kinesins” as well as genes belonging to greenyellow module in “Interferon alpha/beta signaling”, “Cytokine Signaling in Immune system”, “Interferon gamma signaling”, “Host-pathogen interaction of human corona viruses - Interferon induction”, “Antiviral mechanism by IFN-stimulated genes”, “ISG15 antiviral mechanism”, “Type II interferon signaling (IFNG)”, “NOD-like receptor signaling pathway”, “Type I Interferon Induction and Signaling During SARS-CoV-2 Infection”, “SARS-CoV- 2 Innate Immunity Evasion and Cell-specific immune response”, “SARS coronavirus and innate immunity”, “Non-genomic actions of 1,25 dihydroxyvitamin D3”, “Type III interferon signaling”, “Adaptive Immune System”, “IL-10 Anti-inflammatory Signaling Pathway”, “Class I MHC mediated antigen processing & presentation”, “Pathways of nucleic acid metabolism and innate immune sensing” **Supplementary Table 6**. As a whole, cyan DE_Smodule is related to cell dysregulation and lightyellow DE_Smodule corresponds to innate immune suppression.

### Classifier genes and miRNAs between severe and non-severe COVID-19

**Figure 6:**
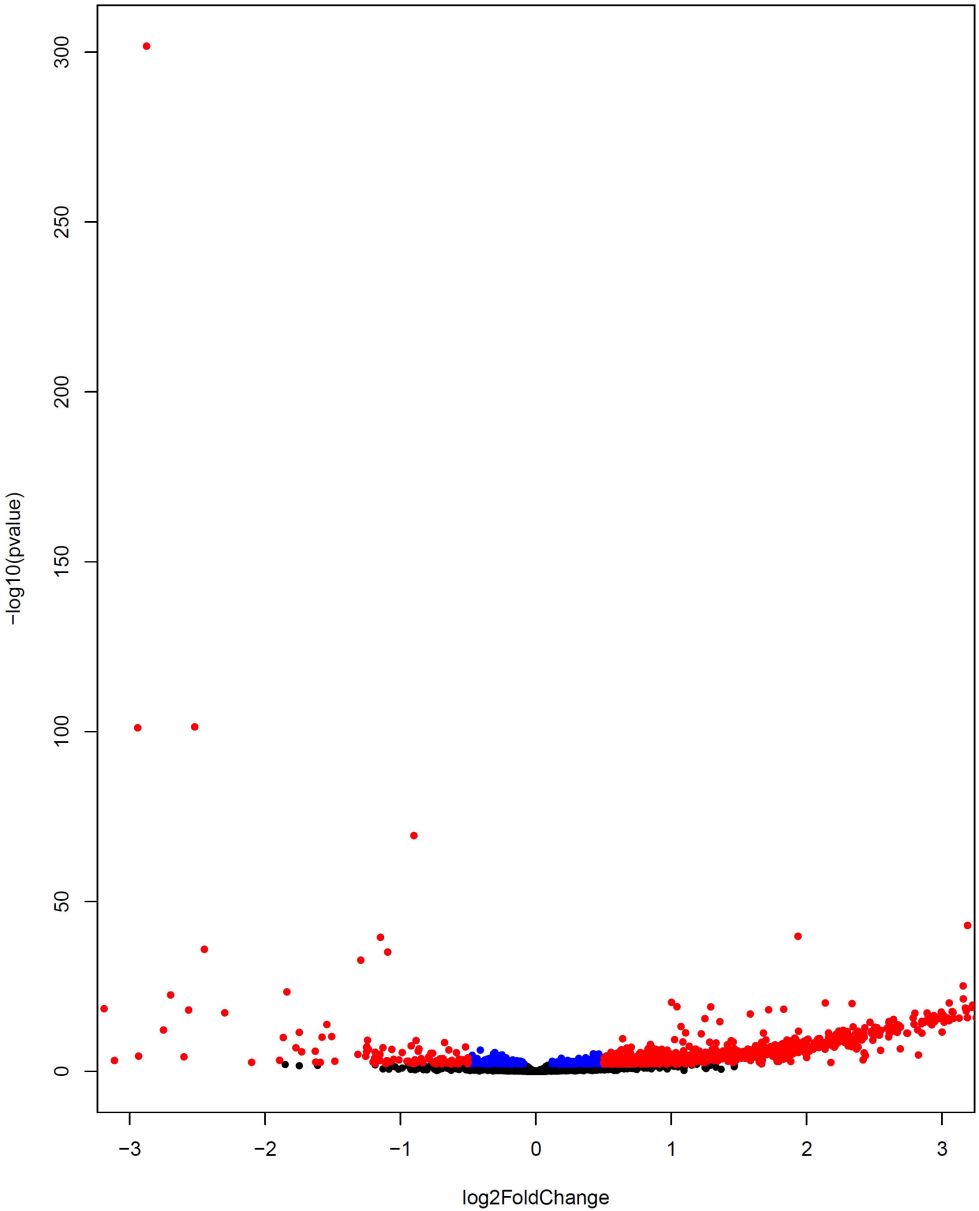
Volcano plot indicating differential gene expression between severe COVID-19 and non-severe COVID-19 subjects. The upregulated and downregulated DEGs are displayed by colors of red and blue, respectively. The non-DEGs are depicted by the black color.

**Figure 7:**
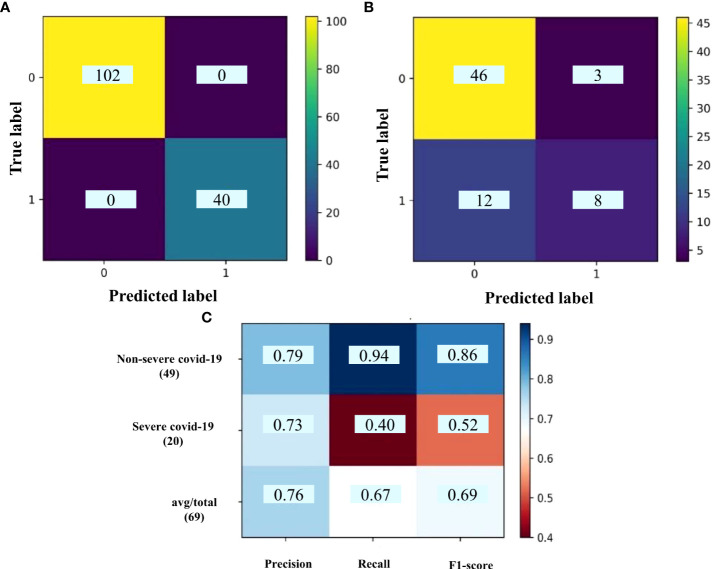
The confusion matrix of **(A)** train set and **(B)** test set; **(C)** classification report for DE_Smodules of brown. The confusion matrix shows the number of true and predicted label in non-severe COVID-19 (label 0) and severe COVID-19 (label 1) subjects. The classification report demonstrates the precision, recall, and F1-score for each group.

The same WGCNA and DEG analyses were performed to determine the related gene groups for severe and non-severe COVID-19 samples. The WGCNA was performed by adjusting the power β to 4. **Figure S6** indicates the cluster dendrogram and modules before and after merging modules. As a result, 11 modules were identified (**Supplementary Table 7**). **Figure S7** demonstrates the module-trait relationships for healthy; and severe and non-severe COVID-19 conditions. Considering p-value< 0.05 and correlation>0.25, module yellow has a remarkable correlation with non-severe COVID-19, black with severe COVID-19, and brown with opposite correlation values with both conditions (Smodules). The preservation of these modules in the external test dataset was also confirmed through module preservation analysis (**Supplementary Table 8**). A total of 504 DEGs between severe and non-severe COVID-19 were determined considering Benjamini-Hochberg adjusted p-value <0.05 and |logFC|=2 genes (**Supplementary Table 9**), of which 477 genes were upregulated and 27 were downregulated as indicated in the volcano plot (**Figure 6**). Afterward, common genes between co-expressed genes in each module and DEGs (DE_Smodule) were explored (**Supplementary Table 10**). The yellow, brown, and black DE_Smodules were used as train input for RFE-SVM. Among them, module brown showed the acceptable accuracy for the train (0.93) and test (0.85) sets as well as other classification parameters (**Table 4**; **Figure 7**; **Figures S8, S9**) with 25 selected features including *ANKRD20A8P*, *CHST3*, *DNM1P51*, *DUX4*, *FAM201B*, *GAD2*, *GAN*, *HOXB13*, *MAB21L4*, *MTCYBP11*, *MTND4P22*, *MTND5P24*, *OR1D2*, *OR52A1*, *OR6U2P*, *OR7E25P*, *PBOV1*, *PIGFP2*, *PTPN5*, *REXO1L1P*, *RHOXF2B*, *RPL35P3*, *TNFRSF6B*, *TPM4P1*, and *VWC2*.

**Table 4:** Accuracy and number of features when genes in DE_Smodules were used.

Module	Accuracy
Yellow	Train: 1 (0.00) Test: 0.75 Number of features: 1
Brown	Train: 0.93 (0.08) Test: 0.85 Number of features: 25
Black	Train: 1 (0.00) Test: 0.78Number of features: 1

**Figure 8:**
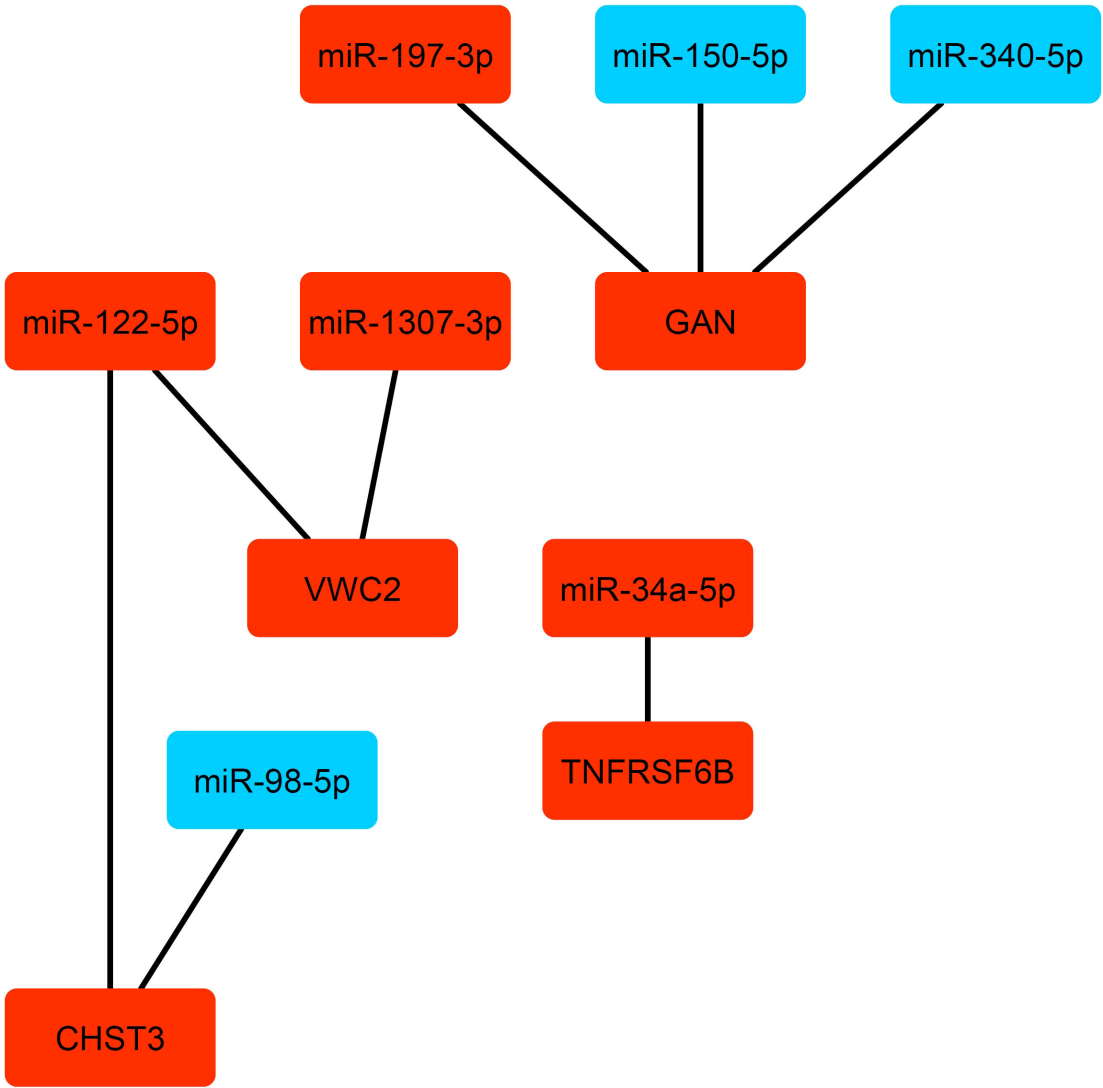
The miRNAs-target gene network. The up-regulated and down-regulated miRNA and genes are depicted by red and blue colors, respectively.

These genes were mostly enriched in the metabolic pathways like Alanine, aspartate and glutamate metabolism; beta-alanine metabolic; butanoate metabolic; and chondroitin sulfate biosynthesis. We further identified 95 differentially expressed miRNA (DEmiRNAs) considering Benjamini-Hochberg-adjusted p-value <0.05 between severe and non-severe conditions (**Supplementary Table 11**). In order to identify the experimentally validated genes for the mentioned DEmiRNAs, miRTarBase database was explored. As a result, a total of 58 miRNAs was determined (**Supplementary Table 12**, sheets 1,2). Then, the common target genes with 25 identified genes as classifiers were found. The resulting miRNAs-target network is depicted in **Figure 8**. From this analysis, miR-34a-5p, miR-122-5p, miR-197-3p, miR-122-5p, miR-1307-3p were upregulated; and miR-98-5p, miR-150-5p, miR-340-5p were downregulated. They target genes comprising *GAN*, *VWC2*, *TNFRSF6B*, and *CHST3.* The miRNAs actually suppress gene expression. However, the network between genes and miRNA is complicated in the human body. Therefore, such subnetworks containing the up-regulated miRNA and genes are not unexpected (13). These miRNAs and their target actually determine the destiny of SARS-CoV-2 infection toward severe or non-severe COVID-19. Therefore, they could be noted for the design of proper treatment.

## Discussion

The rapid universal dispersion of COVID-19 obligates the survey on the dysregulation in the molecular factors due to the SARS-CoV-2 infection. Therefore, the dysregulated genes can be followed to find the possible pathogenesis mechanism as well as therapeutic and diagnostic targets. In this study, we performed sequential steps to identify the classifier genes that differentiate Covid-19 patients from healthy ones. To this end, WGCN and DEG analyses were employed to select the biologically important features (filter phase) and also the dimensional reduction of the data. Afterward, RFECV-SVM as an efficient approach was used for finding the classifiers and optimizing their performance (wrapper phase). Therefore, the combination of the abovementioned approaches led to a powerful biologically information-based machine learning for the identification of biomarker classifiers. It was also used for classification of non-severe Covid-19 from severe Covid-19 samples. However, due to the same origin of these two states of Covid-19, the accuracy was not as well as that obtained for classification of the healthy and Covid-19 subjects and was near to overfitting. It may also due to the fact this module has high correlation with opposite sign with both conditions. The accuracy and other machine learning parameters revealed two significant modules with 5 and 84 gene classifiers for Covid-19 versus healthy subjects. The enrichment analysis disclosed the remarkable roles of some of these genes in innate immune suppression and cell dysregulation. Given the fact that the samples used for gene expression profiling were whole blood or PBMCs, we discussed the obtained genes in this regard. The genes in the cyan and greenyellow DE_Smodules are involved in immune response, particularly innate immunity and cellular mechanisms such as DNA replication or protein export.Moreover, the mechanisms related to Vitamin D and the adaptive immune system were also activated in accordance with our previous report (40). The most important dysregulated pathways by genes in greenyellow DE_Smodule are Interferons response pathways by the function of *USP18*, *RSAD2*, *UBE2L6*, *GBP4*, *DDX58*, *GBP5*, *OASL*, *GBP1*, *GBP3*, *OAS2*, *OAS3*, *XAF1*, *TRIM6*, *IFI27*, *TRIM14*, *IFI6*, *IFIT2*, *EIF2AK2*, *IFIT1*, *IFIT3*, *TRIM5*, *STAT1*, *STAT2*, *HERC5*, and *MX1*. Interferons are one the main defense mechanism against most viral infections such as hepatitis C, herpes simplex virus, measles as well as respiratory viruses including influenza and coronaviruses (41–43). Therefore, the obtained results are not surprising for the SARS-CoV-2 infection. These genes are noteworthy due to their importance in developing COVID-19 and also in the pathogenesis, prognosis, diagnosis, and treatment of the disease. However, it is important that these changes are in the host’s favor and dysregulation of interferon’s responses is one of the main mechanisms for viruses’ evasion from innate immune responses. It reveals the substantial roles of these responses in the control of viral infection (43). The dysregulation of all three types of interferons (I, II, III) in COVID-19 patients reveals the effect of infection on the common genes of these cytokines (such as *STAT1*, *STAT2*). It also confirms the particular status of interferons mechanism in the SARS-CoV-2 infection so that it can be the main mechanism that the virus employs to escape from the immune system (44). Furthermore, “type I interferon induction and signaling during SARS-CoV-2 infection” pathway were enriched in this study which might support this assertion. It has been recently reported that interferon therapy could decrease the time to clinical improvement in COVID-19 patients (45) while there are also contradicting reports about the time of therapy (46). Since multiple genes are involved in the interferons production and their functions, the interpretation of the effect of interferon therapy could be complicated (43).

This complexity is more difficult considering the effect of viral infection on many of these genes. SARS-CoV-2 can also affect these genes (44). As a whole, the reports regarding the lack of positive impact of interferons on COVID-19 are not necessarily interpreted as the insignificance roles of them in the progression of COVID-19. The results of this study also highlight the role of adaptive response including interleukin-10 (IL-10) response, interferon gamma, antigen processing and presentation (*TRIM69*, *FBXO6*, *UBE2L6*, *DTX3L*, *HERC6*, *HERC5*). Since the role of the immune system in the control of disease and its severity has been affirmed, these dysregulated genes and pathways can be in favor or detriment of the host depending on the stage of the disease. According to the results of our study and previous reports, both arms of the immune system (innate and adaptive immunity) have a critical role in the infection fate, so immunomodulation especially in a suitable time of disease can be influential. As previously reported, one of the important immunomodulators is vitamin D (47, 48). Several clinical trials have been performed to survey the effect of vitamin D on COVID-19. Considering the various functions of immunomodulation including boosting antiviral immunity and decreasing inflammation, vitamin D can be a proper approach for COVID-19 treatment (40). Furthermore, due to the high prevalence of vitamin D deficiency in the human population, the prescription of vitamin D as a preventive approach is also sensible. The level of vitamin D may also be beneficial in the effectiveness of vaccines. Our study also confirms the significance of vitamin D pathways with functional roles of RSAD2, OAS2, IFI44L, STAT1, and STAT2 in the pathways of non-genomic actions of 1,25 dihydroxyvitamin D3.

As mentioned before, the selected genes in the cyan module are involved in the cellular mechanisms especially DNA replication, intracellular transmission, and cell division. These pathways were enriched by CD38, MCM6, SEC11C, and K1FC1. Among them, CD38 has been mostly investigated in the immune response. It involves signal transduction, adhesion, and calcium signaling. Moreover, it has a critical role in inflammation and also can be increased by the effect of other inflammatory factors (49). Therefore, the dysregulation of CD38 in COVID-19 can have a role in the control of infection and immunopathogenesis. Moreover, the dysregulation of *SEC11C* has been reported in COVID-19 (50). However, its function has not been discussed. Although due to the diversity of cells in the blood like Treg, Breg, TCD8+, TCD4+, B cells, etc., the dysregulation of these genes leads to different effects on the cells and types of responses including inflammatory and anti-inflammatory immune responses. However, they could be substantial and potential targets for good and poor prognosis as the early identification of cases experiencing severe disease is very vital.

The function of selected genes in cyan and greenyellow modules as the classifiers shows that immune response has a substantial role in the destiny of infection. Furthermore, due to the various outcomes of infection, the role of host genetics might be highly important. Therefore, the investigation of single nucleotide polymorphisms (SNPs) in these genes and their roles in the disease prognosis, and also the therapeutic roles of interferons and CD38 are proposed. The severity of COVID-19 with unclear major reasons could be varying from mild to severe among the population (51). Further analysis of the miRNA-target gene network revealed eight miRNAs including miR-34a-5p, miR-122-5p, miR-197-3p, miR-122-5p, miR-1307-3p, miR-98-5p, miR-150-5p, and miR-340-5p that have different expressions in the severe and non-severe (mild and moderate) COVID-19.

miR‐98‐5p is an oestrogen‐responsive miRNA that can attach and suppress the expression of IL‐6 and influence other proinflammatory cytokines like TNF‐α, and interleukin-1β (52). A distinguished incidence of intussusceptive angiogenesis was also observed in the COVID-19 patients (53). In agreement with this observation, miR-122-5p promotes angiogenesis and is a strong pro-angiogenic factor that actuates vascular endothelial growth factor signaling (54). MiR-122-5p and miR‐98‐5p target *CHST3* which is involved in cell adhesion through synthesizing chondroitin 6-sulfate (55). The up-regulation of some cell adhesion molecules in non-severe and dramatically in severe COVID-19 have been reported and their contributions to coagulation dysfunction have been suggested (56). Another identified DEmiRNA is miR-1307-3p whose its higher expression may result in a decrease in SARS-CoV-2 replication through binding to the 3′ UTR site of the SARS-CoV-2 genome (57). It targets *VWC2* which may have a role in cell adhesion. We also found a higher expression of *CHST3* and *VWC2* in severe samples versus non-severe ones which confirms the aforementioned claims.

The encoded protein GAN has a function in neurofilament architecture and mediates the ubiquitination and degradation of some proteins. Ubiquitination represses replication by targeting viral proteins for degradation and inciting innate antiviral signaling pathways. It also boosts replication by simplifying virion disassembly and viral entry (58, 59). *GAN* is targeted by miR-150-5p, miR-340-5p, and miR-197-3p. The downregulation of miR-340-5p and miR-150-5p can be an antiviral defense mechanism of host cells by up-regulating *GAN* as has been observed in influenza A and other RNA virus infections (60, 61). Moreover, miR- 197-3p has an anti-inflammatory effect and its up-regulation may also be because of the same mechanism of the two above-mentioned miRNAs (62).

According to these observations, it seems that in the severe condition of COVID-19, the antiviral defense mechanisms are more activated. These results are in the line with the role of immune responses in the pathogenesis of COVID-19 as previously reported (51). MiR-34a-5p was also determined as DEmiRNA. It regulates some mRNA targets involved in viral diseases, endothelial, and inflammatory signaling pathways (63). It targets *TNFRSF6B* which encodes a protein that has a regulatory function in repressing LIGHT- and FasL-mediated cell death. The cell death pathways have critical roles in modulating the pathogenesis of disease after viral infection (64). Primary infection and cell death are two major factors that affect the disease course in COVID-19. Host genetics, immune response, and environmental factors can also contribute to determining infection outcomes (64–67). Moreover, the classifier genes between severe and non-severe COVID-19 were enriched in several metabolic pathways. This indicates a remarkable activity of mitochondrial during COVID-19 in agreement with recent mass spectrometer reports (68, 69). Therefore, metabolic pathways and metabolites are key dysregulated pathways and factors that probably determine the fate of SARS- CoV-2 infection. Our study has some limitations. We integrated several datasets containing samples of Covid-19 patients from different regions worldwide. It may affect the result of the analysis, however, we tried to perform rigorous preprocessing data analysis to remove outlier samples and genes. Moreover, we did not consider the variants of Covid-19 because the reference datasets did not mention it.

## Conclusion

In summary, we systematically explored the major functional players with a highly accurate diagnostic power to classify the COVID-19 vs. healthy as well as severe vs. non-severe COVID- 19 subjects. The results revealed that the genes involved in cell dysregulation and interruption of the immune system are the main classifiers between COVID-19 vs. healthy. Moreover, metabolic pathways should be considered as the major pathways to possibly decline the effect of SARS- CoV-2 infection.

## Data availability statement

The original contributions presented in the study are included in the article/**Supplementary Material**. Further inquiries can be directed to the corresponding authors.

## Author contributions

MGZ and EA performed bioinformatics and statistical analysis. MGZ and MT-R interpreted the results and wrote the manuscript. RE supervised the study. All authors approved the final manuscript.

## Acknowledgments

Many thanks to University of Isfahan to support this study.

## Conflict of interest

The authors declare that the research was conducted in the absence of any commercial or financial relationships that could be construed as a potential conflict of interest.

## Publisher’s note

All claims expressed in this article are solely those of the authors and do not necessarily represent those of their affiliated organizations, or those of the publisher, the editors and the reviewers. Any product that may be evaluated in this article, or claim that may be made by its manufacturer, is not guaranteed or endorsed by the publisher.

## Supplementary material

Supplementary Table 1 List of genes in each identified module from the analysis of COVID-19 vs. healthy subjects.

Supplementary Table 2 The results of module preservation analysis for the analysis of COVID-19 vs. healthy subjects.

Supplementary Table 3 List of differentially expressed genes between COVID-19 and healthy subjects.

Supplementary Table 4 Common genes between co-expressed genes in Smodules and DEGs (DE_Smodules).

Supplementary Table 5 The selected classifier genes in DE_Smodules of cyan and lightyellow.

Supplementary Table 6 List of enriched pathways by the DE_Smodules of cyan and lightyellow.

Supplementary Table 7 List of genes in each identified module from the analysis of severe and non-severe COVID-19.

Supplementary Table 8 The results of module preservation analysis for the analysis of severe vs. non-severe COVID-19 subjects.

Supplementary Table 9 List of differentially expressed genes between severe and non- severe COVID-19.

Supplementary Table 10 Common genes between co-expressed genes in Smodules and DEGs (DE_Smodules) (severe and non-severe modules).

Supplementary Table 11 List of differentially expressed miRNA between severe and non-severe subjects.

Supplementary Table 12 Common miRNA between DE_miRNA and miRTarBase database (sheet 1) and common miRNAs-target.genes (sheet 2).
