# Supplementary material for: Exploration of blood−derived coding and non-coding RNA diagnostic immunological panels for COVID-19 through a co-expressed-based machine learning procedure
Source: Front Immunol. 2022 Nov 3;13:1001070. doi: 10.3389/fimmu.2022.1001070 (PMC9670818; doi:10.3389/fimmu.2022.1001070)
Supplement: Supplementary file 1 [file DataSheet_1.docx]

**
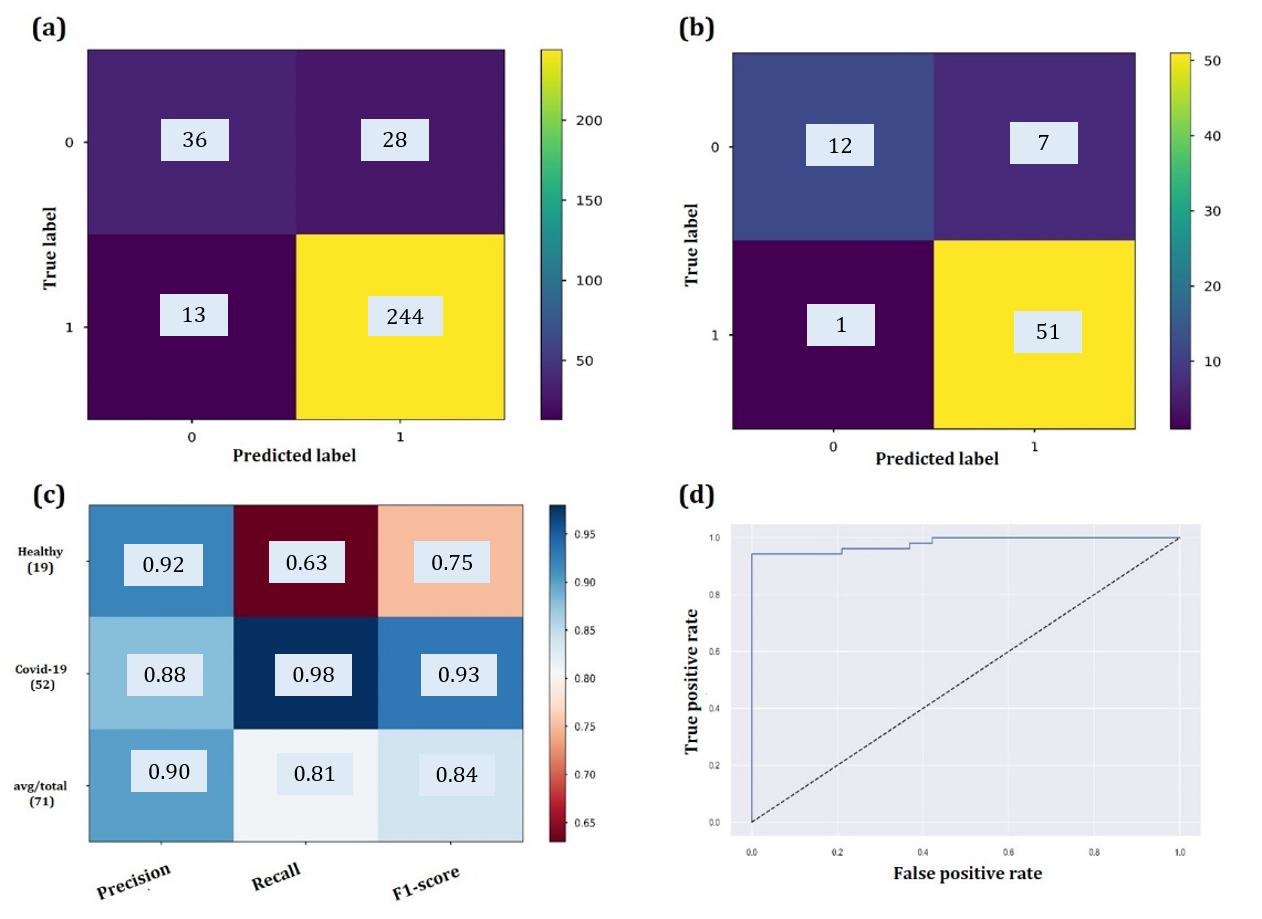
**

**Figure S1.** The confusion matrix of (a) train set and (b) test set; (c) classification report; (d) ROC curve of cyan Smodule. The confusion matrix shows the number of true and predicted label in non-severe COVID-19 (label 0) and severe COVID-19 (label 1) subjects. The classification report demonstrates the precision, recall, and F1-score for each group.


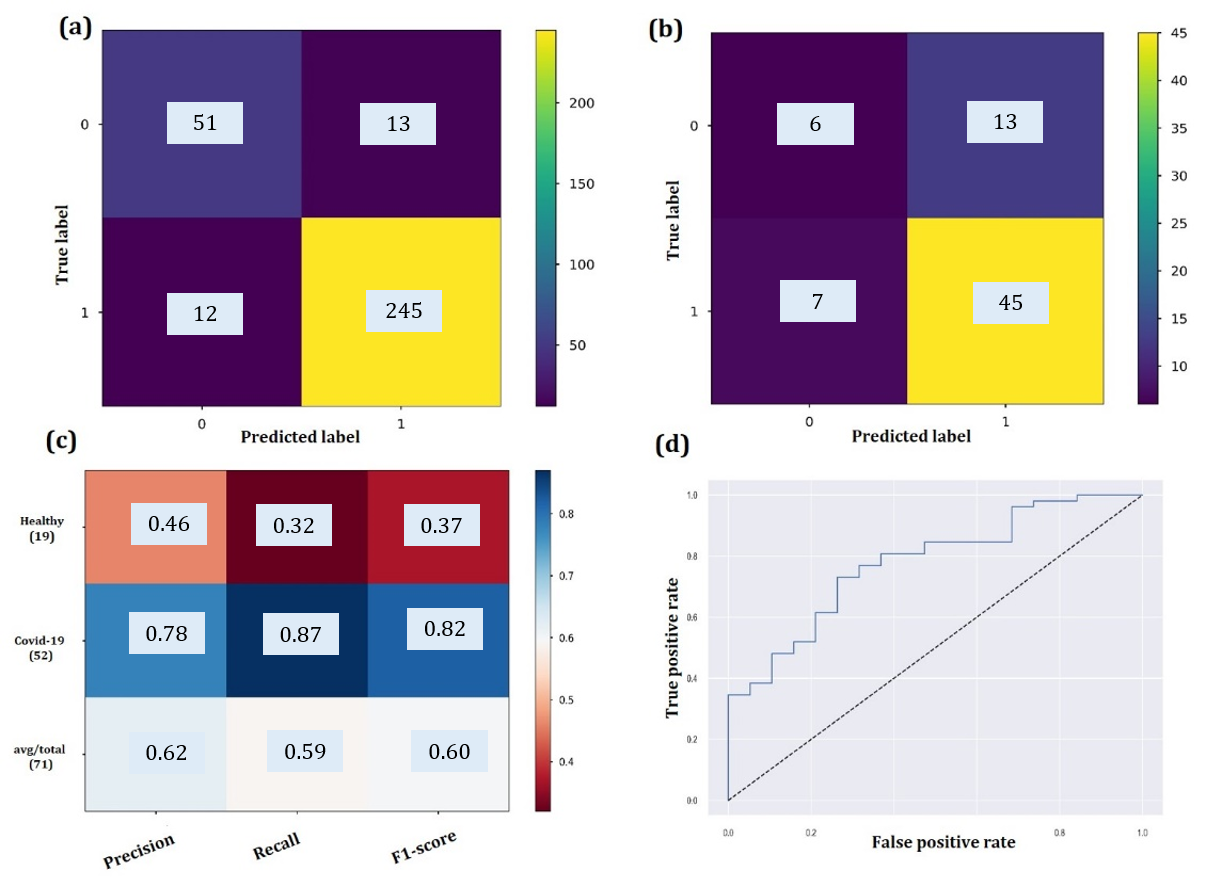


**Figure S2.** The confusion matrix of (a) train set and (b) test set; (c) classification report; (d) ROC curve of darkturquoise Smodule. The confusion matrix shows the number of true and predicted label in non-severe COVID-19 (label 0) and severe COVID-19 (label 1) subjects. The classification report demonstrates the precision, recall, and F1-score for each group.


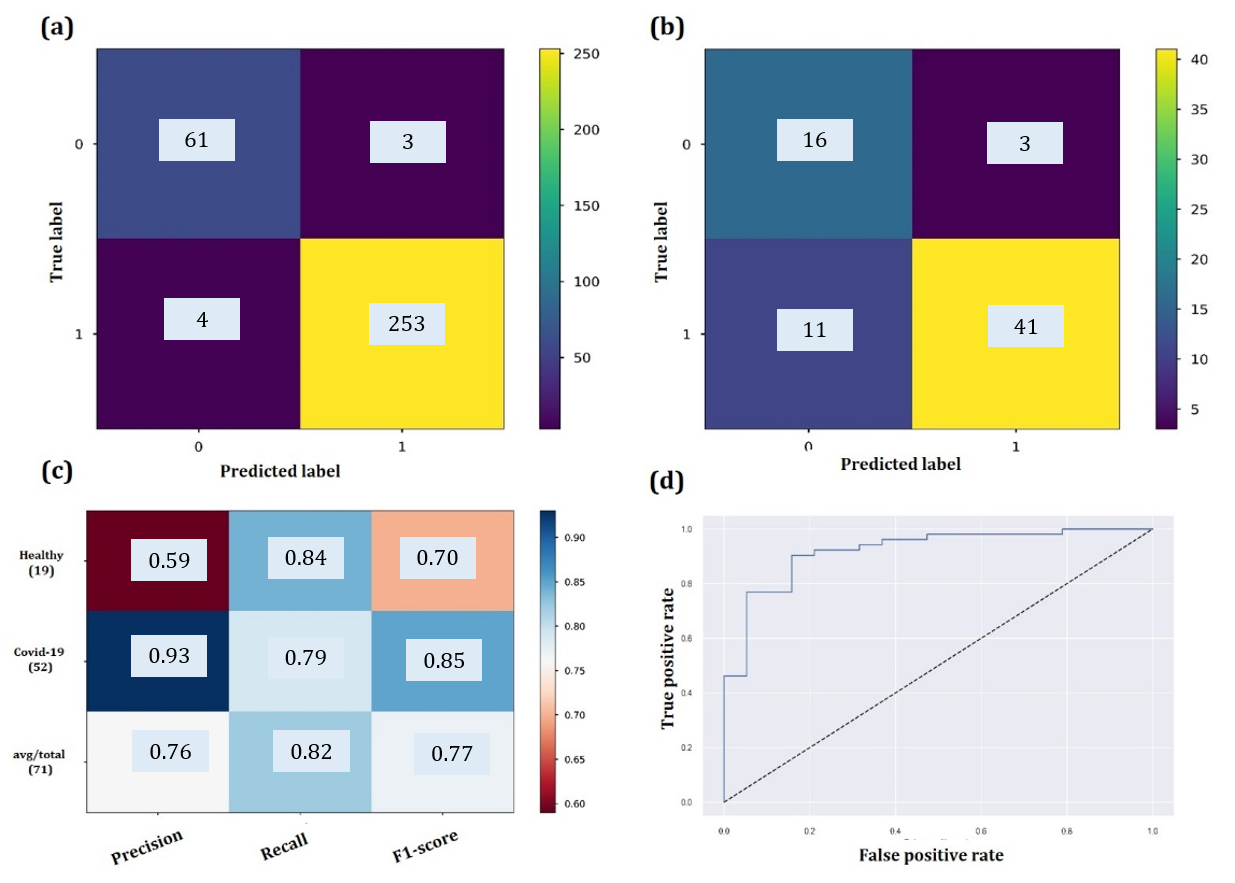


**Figure S3.** The confusion matrix of (a) train set and (b) test set; (c) classification report; (d) ROC curve of lightyellow Smodule. The confusion matrix shows the number of true and predicted label in non-severe COVID-19 (label 0) and severe COVID-19 (label 1) subjects. The classification report demonstrates the precision, recall, and F1-score for each group.


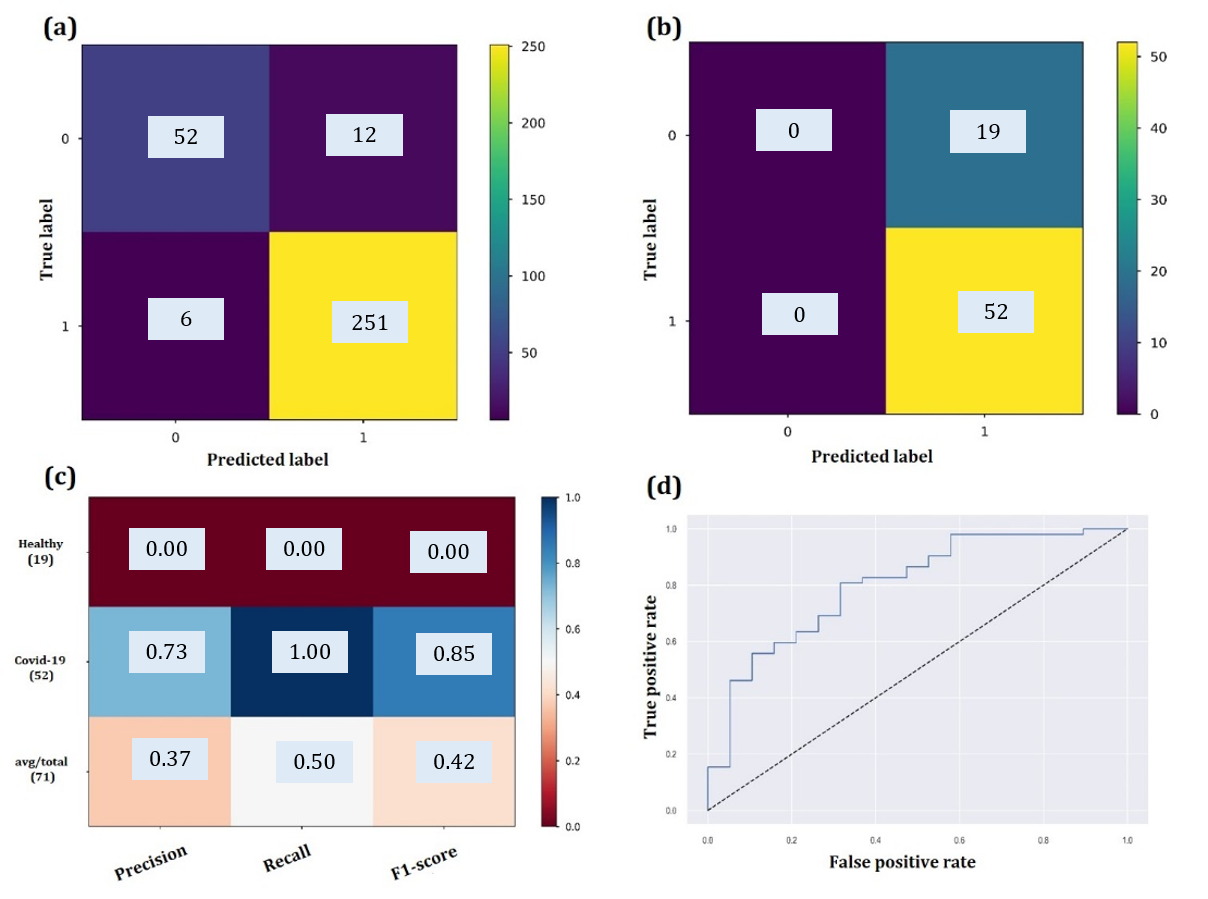


**Figure S4.** The confusion matrix of (a) train set and (b) test set; (c) classification report; (d) ROC curve of midnightblue Smodule. The confusion matrix shows the number of true and predicted label in non-severe COVID-19 (label 0) and severe COVID-19 (label 1) subjects. The classification report demonstrates the precision, recall, and F1-score for each group.


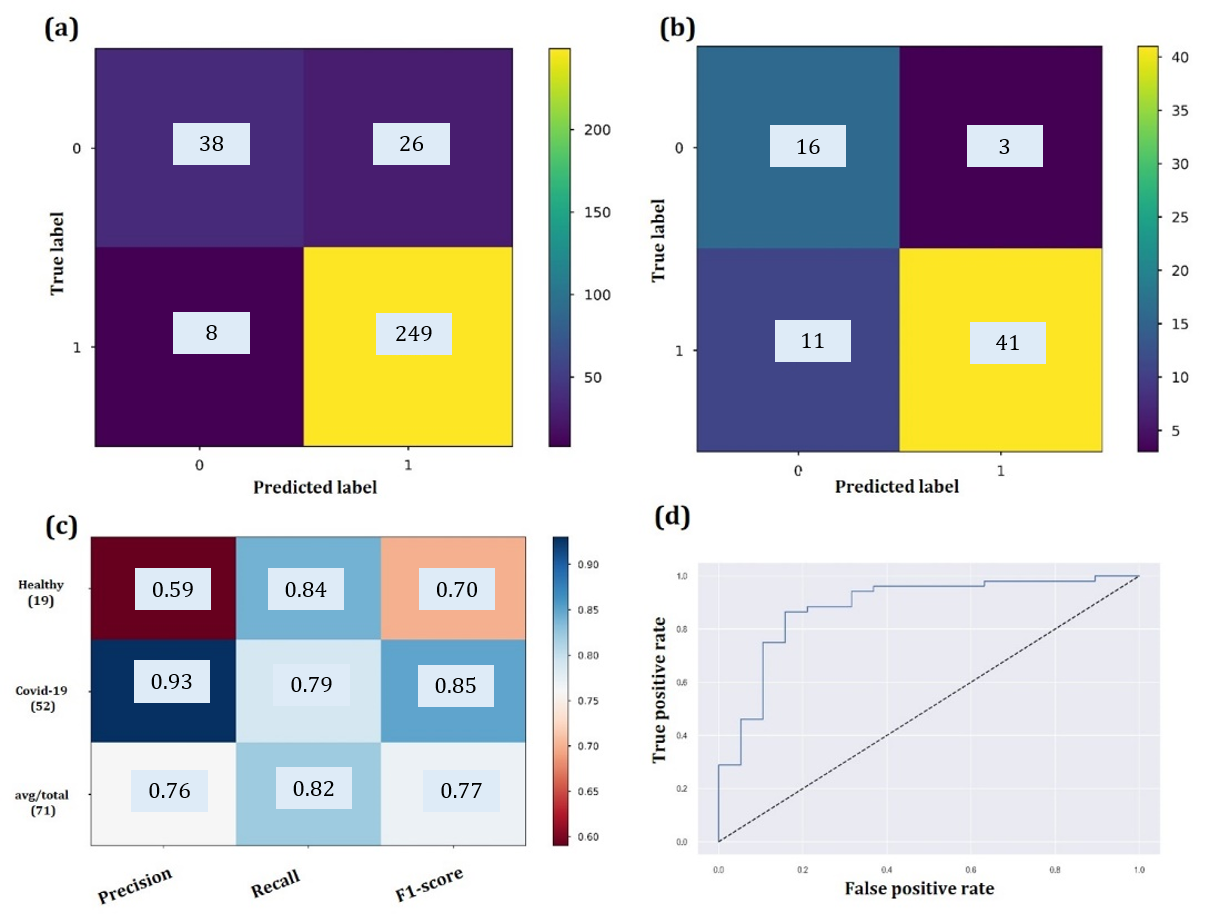


**Figure S5.** The confusion matrix of (a) train set and (b) test set; (c) classification report; (d) ROC curve of orange Smodule. The confusion matrix shows the number of true and predicted label in non-severe COVID-19 (label 0) and severe COVID-19 (label 1) subjects. The classification report demonstrates the precision, recall, and F1-score for each group.


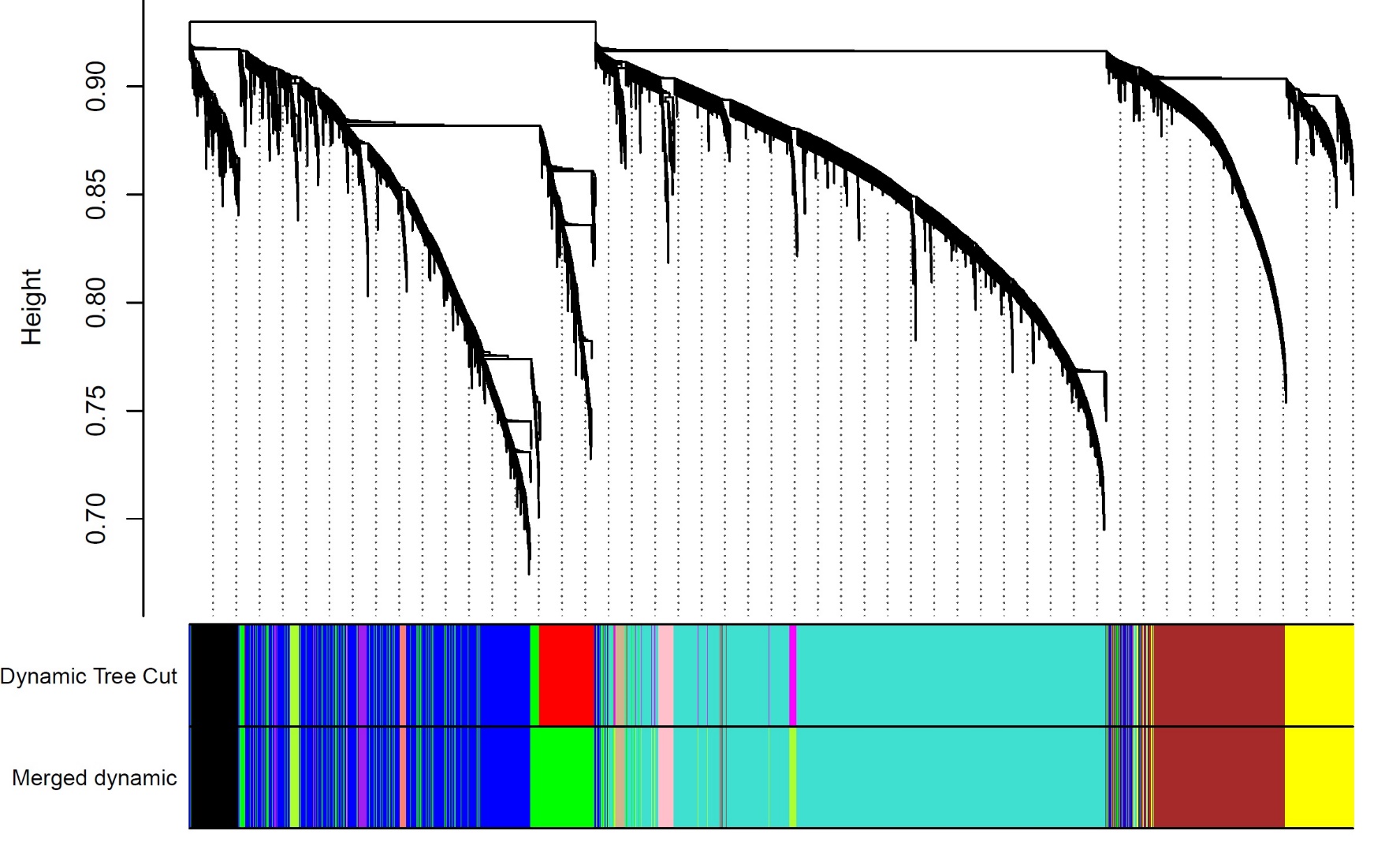


**Figure 6S.** Cluster dendrogram and modules before and after merging. The branches of the dendrogram cluster demonstrate the compact interconnected and also highly co-expressed genes; Each color demonstrates an individual module.


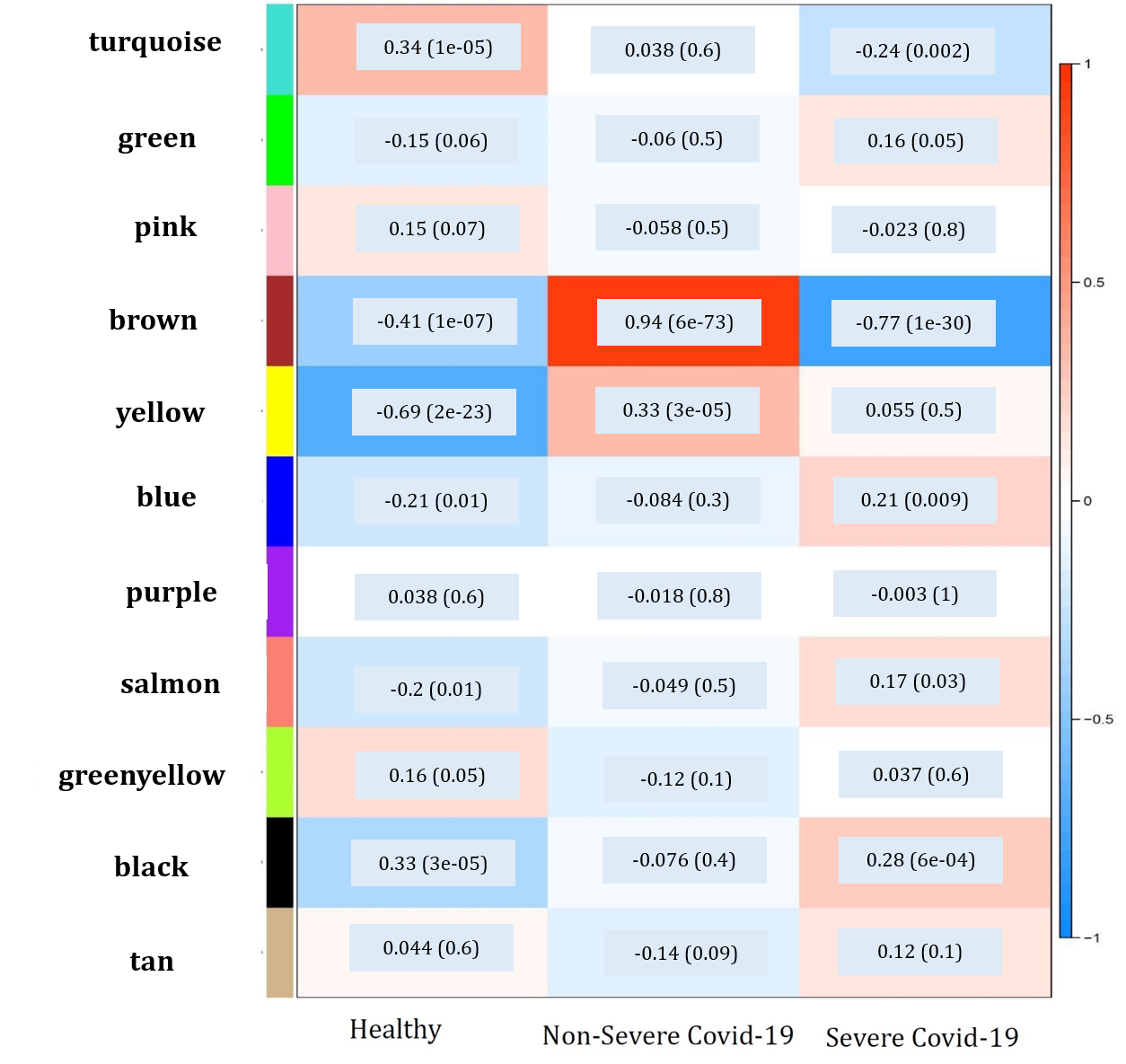


**Figure S7.** The module-trait relationships for healthy; severe and non-severe Covid-19, in which the correlation between modules and each condition are specified.

**tan**

**black**

0.28 (6e-04)

-0.076 (0.4)

-0.33 (3e-05)


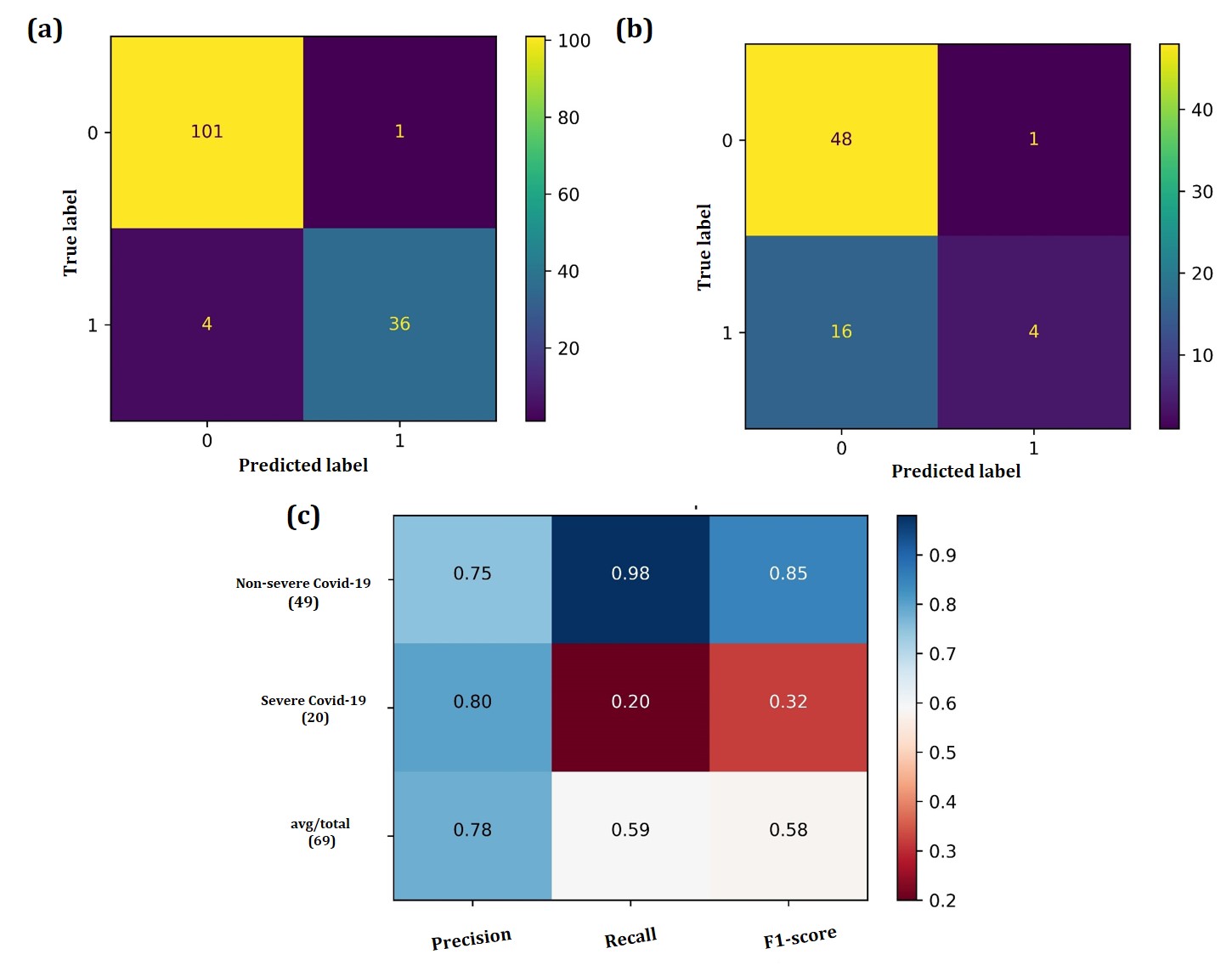


**Figure S8.** The confusion matrix of (a) train set and (b) test set; (c) classification report; (d) ROC curve of yellow DE_Smodule. The confusion matrix shows the number of true and predicted label in non-severe COVID-19 (label 0) and severe COVID-19 (label 1) subjects. The classification report demonstrates the precision, recall, and F1-score for each group


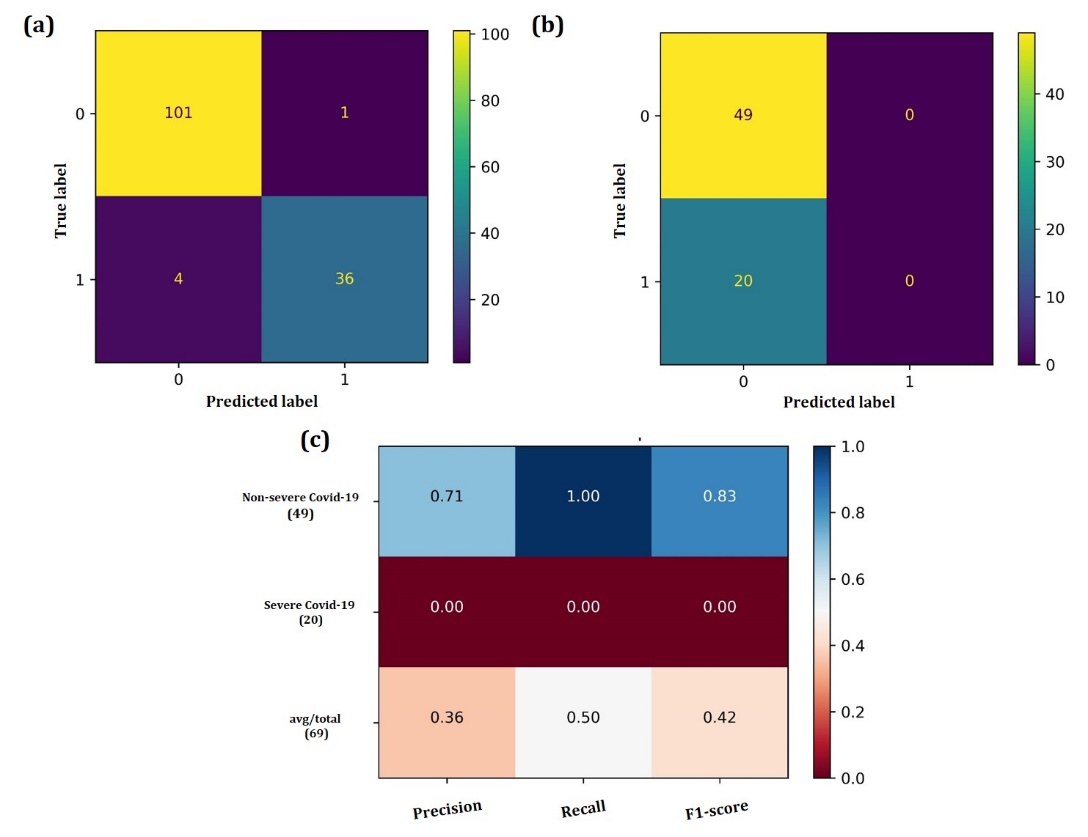


**Figure S9.** The confusion matrix of (a) train set and (b) test set; (c) classification report; (d) ROC curve of black DE_Smodule. The confusion matrix shows the number of true and predicted label in non-severe COVID-19 (label 0) and severe COVID-19 (label 1) subjects. The classification report demonstrates the precision, recall, and F1-score for each group.
