# Supplementary material for: Exploration of blood−derived coding and non-coding RNA diagnostic immunological panels for COVID-19 through a co-expressed-based machine learning procedure
Source: Front Immunol. 2022 Nov 3;13:1001070. doi: 10.3389/fimmu.2022.1001070 (PMC9670818; doi:10.3389/fimmu.2022.1001070)
Supplement: Supplementary file 2 [file DataSheet_2.docx]

### WGCNA Codes for determining the co-expressed modules

library(limma)

library(WGCNA)

library(cluster)

library(flashClust)

library(preprocessCore)

# Loading data

Normal=read.csv("Normal.csv",row.names = 1)

Covid=read.csv("Covid.csv",row.names = 1)

Data=cbind(Normal, Covid)

datExpr=as.data.frame((t(Data)))

# Filtering outlier genes

gsg = goodSamplesGenes(datExpr, verbose = 3);

gsg$allOK

if (!gsg$allOK)

{

# Optionally, print the gene and sample names that were removed:

if (sum(!gsg$goodGenes)>0)

printFlush(paste("Removing genes:", paste(names(datExpr)[!gsg$goodGenes], collapse = ", ")));

if (sum(!gsg$goodSamples)>0)

printFlush(paste("Removing samples:", paste(rownames(datExpr)[!gsg$goodSamples], collapse = ", ")));

# Remove the offending genes and samples from the data:

datExpr = datExpr[gsg$goodSamples, gsg$goodGenes]

}

sampleTree = hclust(dist(datExpr), method = "average");

pdf(file = "1_Sample clustering.pdf", width = 12, height = 9);

par(cex = 0.6);

par(mar = c(0,4,2,0))

plot(sampleTree, main = "Sample clustering to detect outliers", sub="", xlab="", cex.lab = 1.5, cex.axis = 1.5, cex.main = 2)

dev.off()

# Determining cluster under the line

clust = cutreeStatic(sampleTree, cutHeight = 700, minSize = 10)

table(clust)

# clust 1 contains the samples we want to keep.

keepSamples = (clust==1)

datExpr = datExpr[keepSamples, ]

nGenes = ncol(datExpr)

nSamples = nrow(datExpr)

datExpr=datExpr

#Network construction and module detection

# Choose a set of soft-thresholding powers

powers = c(c(1:12), seq(from = 14, to=20, by=2))

# Call the network topology analysis function

sft = pickSoftThreshold(datExpr, powerVector = powers, verbose = 5)

# Plot the results:

pdf(file="2_Soft Threshold and Mean Connectivity.pdf", height=10, width=18)

par(mfrow = c(1,2));

cex1 = 0.9;

# Scale-free topology fit index as a function of the soft-thresholding power

plot(sft$fitIndices[,1], -sign(sft$fitIndices[,3])*sft$fitIndices[,2], xlab="Soft Threshold (power)",ylab="Scale Free Topology Model Fit,signed R^2",type="n",main = paste("Scale independence"));

text(sft$fitIndices[,1], -sign(sft$fitIndices[,3])*sft$fitIndices[,2],

labels=powers,cex=cex1,col="red");

# this line corresponds to using an R^2 cut-off of abline(h=0.8,col="red")

# Mean connectivity as a function of the soft-thresholding power

plot(sft$fitIndices[,1], sft$fitIndices[,5],

xlab="Soft Threshold (power)",ylab="Mean Connectivity", type="n",

main = paste("Mean connectivity"))

text(sft$fitIndices[,1], sft$fitIndices[,5], labels=powers, cex=cex1,col="red")

dev.off()

#Set softPower

softPower = optimum power;

adjacency = adjacency(datExpr, type="signed",corFnc="bicor",power = softPower);

# Turn adjacency into topological overlap

TOM = TOMsimilarity(adjacency,TOMType="signed");

dissTOM= 1-TOM

# Hierarchical clustering function

geneTree= hclust(as.dist(dissTOM), method = "average");

# Plot the resulting clustering tree (dendrogram)

pdf(file="3_Gene clustering on TOM-based dissimilarity_step by step.pdf", height=10, width=18)

plot(geneTree, xlab="", sub="", main = "Gene clustering on TOM-based dissimilarity",

labels = FALSE, hang = 0.04);

dev.off()

minModuleSize = 30;

# Module identification using dynamic tree cut:

dynamicMods = cutreeDynamic(dendro = geneTree, distM = dissTOM,

deepSplit = 2, pamRespectsDendro = FALSE,

minClusterSize = minModuleSize);

table(dynamicMods)

# Converting numeric lables into colors

dynamicColors = labels2colors(dynamicMods)

table(dynamicColors)

# Plot the dendrogram and colors underneath

pdf(file="4_Dynamic Tree Cut_Step by Step.pdf", height=10, width=18)

plotDendroAndColors(geneTree, dynamicColors, "Dynamic Tree Cut",

dendroLabels = FALSE, hang = 0.03,

addGuide = TRUE, guideHang = 0.05,

main = "Gene dendrogram and module colors")

dev.off()

# Calculating eigengenes

MEList = moduleEigengenes(datExpr, colors = dynamicColors)

MEs = MEList$eigengenes

# Calculating dissimilarity of module eigengenes

MEDiss = 1-cor(MEs);

# Clustering module eigengenes

METree = hclust(as.dist(MEDiss), method = "average");

# Plotting the result

pdf(file="5_Clustering of module eigengenes_Step by step.pdf", height=15, width=25)

plot(METree, main = "Clustering of module eigengenes",

xlab = "", sub = "")

MEDissThres = 0.05

#Plotting the cut line into the dendrogram

abline(h=MEDissThres, col = "red")

dev.off()

# Merging close modules

merge = mergeCloseModules(datExpr, dynamicColors, cutHeight = MEDissThres, verbose = 3)

# The merged module colors

mergedColors = merge$colors;

# Eigengenes of the new merged modules:

mergedMEs = merge$newMEs;

pdf(file = "6_geneDendro-mergedmodules.pdf", wi = 9, he = 6)

plotDendroAndColors(geneTree, cbind(dynamicColors, mergedColors),

c("Dynamic Tree Cut", "Merged dynamic"),

dendroLabels = FALSE, hang = 0.03,

addGuide = TRUE, guideHang = 0.05)

dev.off()

# Renaming to moduleColors

moduleColors= mergedColors

# Construct numerical labels corresponding to the colors

colorOrder = c("grey", standardColors(100));

moduleLabels = match(mergedColors, colorOrder)-1;

MEs= mergedMEs;

#Renaming to moduleColors

moduleColors = moduleColors

# Constructing numerical labels corresponding to the colors

colorOrder = c("grey", standardColors(50));

moduleLabels = match(moduleColors, colorOrder)-1;

MEs = mergedMEs;

save(datExpr,MEs,TOM,dissTOM,geneTree,adjacency,moduleColors, moduleLabels,file="ATLL.RData")

# Calculating topological overlap anew: this could be done more efficiently by saving the TOM

# calculated during module detection, but let us do it again here.

dissTOM = 1-TOMsimilarityFromExpr(datExpr, power = softpower);

# Transform dissTOM with a power to make moderately strong connections more visible in the heatmap

plotTOM = dissTOM^softpower;

# Setting diagonal to NA for a nicer plot

diag(plotTOM) = NA;

sizeGrWindow(9,9)

TOMplot(plotTOM, geneTree, moduleColors, main = "Network heatmap plot, all genes")

nSelect = 2000

#For reproducibility, we set the random seed

set.seed(10);

select = sample(nGenes, size = nSelect);

selectTOM = dissTOM[select, select];

# There's no simple way of restricting a clustering tree to a subset of genes, so we must re-cluster.

selectTree = hclust(as.dist(selectTOM), method = "average")

selectColors = moduleColors[select];

# Open a graphical window

#sizeGrWindow(9,9)

# Taking the dissimilarity to a power, makes the plot more informative by effectively changing

# the color palette; setting the diagonal to NA also improves the clarity of the plot

plotDiss = selectTOM^ softpower;

diag(plotDiss) = NA;

pdf(file="7_Network heatmap plot, selected genes.pdf", height=10, width=18)

TOMplot(plotDiss, selectTree, selectColors, main = "Network heatmap plot, selected genes")

dev.off()

# Recalculating module eigengenes

MEs = moduleEigengenes(datExpr, moduleColors)$eigengenes

# Module eigengenes

MET = orderMEs(cbind(MEs))

# Plot the relationships among the eigengenes

sizeGrWindow(5,7.5);

pdf(file="8_EigengeneNetworks.pdf", height=10, width=18)

plotEigengeneNetworks(MET, "", marDendro = c(0,4,1,2), marHeatmap = c(3,4,1,2), cex.lab = 0.8, xLabelsAngle

= 90)

dev.off()

# Plotting the dendrogram

par(cex = 1.0)

pdf(file="9_Eigengene dendrogram.pdf", height=10, width=18)

plotEigengeneNetworks(MET, "Eigengene dendrogram", marDendro = c(0,4,2,0),

plotHeatmaps = FALSE)

dev.off()

# Plotting the heatmap matrix (note: this plot will overwrite the dendrogram plot)

par(cex = 1.0)

pdf(file="10_Eigengene adjacency heatmap.pdf", height=10, width=18)

plotEigengeneNetworks(MET, "Eigengene adjacency heatmap", marHeatmap = c(3,4,2,2),

plotDendrograms = FALSE, xLabelsAngle = 90)

dev.off()

write.csv(mergedColors,file="mergedColors.csv")

#Exportting of networks to external software

# Recalculating topological overlap

TOM = TOMsimilarityFromExpr(datExpr, power = softpower);

nTop = 500;

# Exportting modules

Modules=unique(moduleColors)

for (i in 1:length(Modules)) {

# module=as.character(Modules[i,])

module=Modules[i]

# Select module probes

probes = names(datExpr)

inModule = (mergedColors==module);

modProbes = probes[inModule];

# Select the corresponding Topological Overlap

modTOM = TOM[inModule, inModule];

dimnames(modTOM) = list(modProbes, modProbes)

# Export the network into an edge list file VisANT can read

IMConn = softConnectivity(datExpr[, modProbes]);

top = (rank(-IMConn) <= nTop)

vis= exportNetworkToVisANT(modTOM[top, top],

file = paste( module, "module.txt", sep=""),

weighted = TRUE,

threshold = 0.02,

probeToGene = data.frame(modProbes, modProbes) )

}

datTraits = read.csv("Traits.csv", row.names=1)

data=datExpr

moduleTraitCor = cor(MEs, datTraits, use = "p");

moduleTraitPvalue = corPvalueStudent(moduleTraitCor, nSamples);

colnames(moduleTraitPvalue) = paste("p.value.", colnames(moduleTraitCor), sep="");

pdf("module-trait_relationships2.pdf", height=10, width=15)

textMatrix = paste(signif(moduleTraitCor, 2), "\n(",

signif(moduleTraitPvalue, 1), ")", sep = "")

dim(textMatrix) = dim(moduleTraitCor)

par(mar = c(6, 8.5, 3, 3))

# Display the correlation values within a heatmap plot

labeledHeatmap(Matrix = moduleTraitCor, xLabels = colnames(datTraits),

yLabels = colnames(MEs), ySymbols = colnames(MEs),

colorLabels =FALSE,colors=blueWhiteRed(50),textMatrix=textMatrix,

setStdMargins = FALSE, cex.text = 0.5, zlim = c(-1,1))

dev.off()

out<-cbind(Module=rownames(moduleTraitCor ), moduleTraitCor, moduleTraitPvalue)

#write out correlations

write.csv(out, "moduleTraitCor.csv")

### SVM-RFECV

import numpy as np

import pandas as pd

import matplotlib.pyplot as plt

import itertools

from matplotlib import rcParams

plt.rc('figure', figsize=(12,7) ) # Image size

import seaborn as sns

import sklearn

from sklearn.svm import SVC

from sklearn.model_selection import StratifiedKFold

from sklearn.feature_selection import RFECV

from sklearn.metrics import confusion_matrix

from sklearn.metrics import accuracy_score

from sklearn.metrics import classification_report

import csv

from sklearn.model_selection import cross_val_score

from sklearn.model_selection import train_test_split

from sklearn.metrics import confusion_matrix, plot_confusion_matrix

#import Data

x_train,x_test,y_train,y_test = pd.read_csv('insert path.csv')

# Linear RFE-SVM classifier

model_Linear_SVM = svm.SVC(kernel='linear', probability=True)

rfecv = RFECV(estimator=model_Linear_SVM, step=1, cv=10, scoring='accuracy') #10-fold cross-validation

rfecv = rfecv.fit(x_train, y_train)

#Number of classifiers

df=rfecv.n_features_

df1=x_train.columns[rfecv.support_]

#Accuracy of Model

scores = cross_val_score(rfecv, x_train, y_train, scoring='accuracy', cv=10, n_jobs=-1)

print('Accuracy of Train: %.3f (%.3f)' % (mean(scores), std(scores)))

y_test_scores_linear=rfecv.predict(x_test)

#Accuracy of Test

y_test_scores_linear1 = pd.DataFrame(y_test_scores_linear)

print('Accuracy of Test: %.3f (%.3f)' % (mean(y_test_scores_linear1), std(y_test_scores_linear1)))

df = classification_report(y_test, y_test_scores_linear)

#def plot_classification_report(classificationReport, title='Classification report', cmap='RdBu'):

def plot_classification_report(classificationReport, cmap='RdBu'):

classificationReport = classificationReport.replace('\n\n', '\n')

classificationReport = classificationReport.replace(' / ', '/')

lines = classificationReport.split('\n')

classes, plotMat, support, class_names = [], [], [], []

for line in lines[1:]: # if you don't want avg/total result, then change [1:] into [1:-1]

t = line.strip().split()

if len(t) < 2:

continue

classes.append(t[0])

v = [float(x) for x in t[1: len(t) - 1]]

support.append(int(t[-1]))

class_names.append(t[0])

plotMat.append(v)

plotMat = np.array(plotMat)

xticklabels = ['Precision', 'Recall', 'F1-score']

yticklabels = ['{0} ({1})'.format(class_names[idx], sup)

for idx, sup in enumerate(support)]

plt.imshow(plotMat, interpolation='nearest', cmap=cmap, aspect='auto')

#plt.title(title)

plt.colorbar()

plt.xticks(np.arange(3), xticklabels, rotation=45)

plt.yticks(np.arange(len(classes)), yticklabels)

upper_thresh = plotMat.min() + (plotMat.max() - plotMat.min()) / 10 * 8

lower_thresh = plotMat.min() + (plotMat.max() - plotMat.min()) / 10 * 2

for i, j in itertools.product(range(plotMat.shape[0]), range(plotMat.shape[1])):

plt.text(j, i, format(plotMat[i, j], '.2f'),

horizontalalignment="center",

color="white" if (plotMat[i, j] > upper_thresh or plotMat[i, j] < lower_thresh) else "black")

#plt.ylabel('Metrics')

#plt.xlabel('Classes')

plt.tight_layout()

sampleClassificationReport =""“ precision recall f1-score support

Healthy

Covid-19

avg / total """

plot_classification_report(sampleClassificationReport)

plt.savefig("image.pdf", dpi=600)

M = confusion_matrix(y_test,y_test_scores_linear1)

tn, fp, fn, tp = M.ravel()

# plotting the confusion matrix

plot_confusion_matrix( rfecv, x_train, y_train)

plt.savefig("image.pdf", dpi=600)

# plotting the confusion matrix

plot_confusion_matrix( rfecv, x_test, y_test)

#plt.show()

plt.savefig("image.pdf", dpi=600)

from sklearn.metrics import roc_curve, roc_auc_score

y_pred_proba = rfecv.predict_proba(np.array(x_test))[:,1]

fpr, tpr, thresholds = roc_curve(y_test, y_pred_proba)

sns.set()

plt.plot(fpr, tpr)

plt.plot(fpr, fpr, linestyle = '--', color = 'k')

plt.xlabel('False positive rate')

plt.ylabel('True positive rate')

AUROC = np.round(roc_auc_score(y_test, y_pred_proba), 2)

plt.savefig("ROC_image.pdf", dpi=600)
